# Blood-based protein biomarkers during the acute ischemic stroke treatment window: a systematic review

**DOI:** 10.3389/fneur.2024.1411307

**Published:** 2024-07-18

**Authors:** Jan Rahmig, Aditya Chanpura, Aaliyah Schultz, Frank C. Barone, Deborah Gustafson, Alison E. Baird

**Affiliations:** ^1^Department of Neurology, State University of New York Downstate Health Sciences University, Brooklyn, NY, United States; ^2^Department of Neurology, University Hospital Carl Gustav Carus, Technical University Dresden, Dresden, Germany

**Keywords:** ischemic stroke, protein-based biomarkers, diagnosis, prediction, blood biomarkers

## Abstract

**Background:**

Rapid and accurate acute ischemic stroke (AIS) diagnosis is needed to expedite emergent thrombolytic and mechanical thrombectomy treatment. Changes in blood-based protein biomarkers during the first 24 h of AIS, the time window for treatment, could complement imaging techniques and facilitate rapid diagnosis and treatment.

**Methods:**

We performed a systematic review according to PRISMA guidelines. MEDLINE, EMBASE, Cochrane Library, and Web of Science databases were searched for eligible studies comparing levels of blood-based protein biomarkers in AIS patients with levels in healthy controls and stroke mimics. Protein biomarkers from the following pathophysiological categories were included: neurovascular inflammation (MMP-9, TNF-alpha), endothelial integrity (VCAM-1, ICAM-1), cell migration (E-Selectin, P-Selectin, L-Selectin), markers of glial and neuronal origin (GFAP, S100, S100B, NSE), and cardiac dysfunction (BNP, NT-proBNP). The literature search was limited to English-language publications before November 7th, 2023.

**Results:**

A total of 61 studies from 20 different countries were identified, which included in total, 4,644 AIS patients, 2,242 stroke mimics, and 2,777 controls. Studies investigating TNF-alpha, MMP-9, VCAM-1, ICAM-1, E-Selectin, L-Selectin, GFAP, NSE, and S100B showed pronounced methodological heterogeneity, making between-study comparisons difficult. However, in 80% of NT-proBNP and BNP studies, and all P-selectin studies, higher biomarker levels were observed in AIS patients compared to healthy controls and/or patients with stroke mimics.

**Conclusion:**

None of the biomarkers included showed sufficient evidence for additional diagnostic benefit for AIS. Comprehensive standardized global multicenter studies are needed to (1) permit comparability, (2) enable valid statements about protein-based biomarkers, and (3) reflect real-world scenarios.

## 1 Introduction

Stroke is the third leading cause of long-term disability and the second leading cause of death worldwide (1). The two major stroke subtypes are ischemic and hemorrhagic stroke, with 87% of all strokes being ischemic (2). The detection of intracerebral hemorrhagic stroke with non-contrast computerized tomography (CT) has a sensitivity approaching 100% when performed within 6 h of stroke symptoms onset (3). However the early detection of acute ischemic stroke (AIS) with CT has a much lower sensitivity, ranging from 57 to 71%, during the first hours after symptom onset (3–5). Although brain magnetic resonance imaging (MRI) has a 79.8% sensitivity for AIS detection in the hyperacute phase, MRI not only takes more time but is also not widely used in emergency or under-resourced settings (6). Rapid and accurate diagnosis is required in the first 24 h of AIS, since patients may be eligible for reperfusion therapy with intravenous recombinant tissue plasminogen activator (rt-PA) within the first 4.5 h, and endovascular therapy (EVT) up to 24 h in some patients with large vessel occlusions (7). In addition, economic and logistical factors including availability of brain MRI or interventional radiology resources for EVT, infrastructure, and fast biomarker detection devices play key roles in acute diagnosis and intervention decisions (8).

Blood-based protein biomarkers could complement the diagnosis of AIS because of their relative ease and rapidity of use, and low cost (9–11). To be clinically applicable, blood-based biomarkers need to be specific, sensitive, and reliable (8). However, no diagnostically accurate protein biomarkers from blood have yet been proven to have the required diagnostic accuracy for clinical application and decision-making in AIS. This may change with the continued and accelerated development of sensitive and accurate assay methods for the detection of blood-based protein biomarkers. For example, compared to Enzyme-linked Immunosorbent Assay (ELISA), Single Molecule Array (SIMOA) provides enhanced measurement resolution to as little as femtogram/ml concentrations. Furthermore, Point-of-care technologies are continually evolving (12–14). SIMOA revitalized the investigation of blood-based glial fibrillary acid protein (GFAP) and matrix-metalloproteinase 9 (MMP-9) (15, 16). With SIMOA and other constantly improving measurement methods such as point of care (POCT)-devices, the question arises as to whether these more sensitive and specific techniques may lead to new insights regarding the utility of circulating protein biomarkers in AIS diagnosis. Furthermore, now that an extended time window for EVT has been achieved in AIS patients suffering from large vessel occlusions (LVO), with large, salvageable ischemic penumbras compared to small cores, validated blood tests could be used in the field to transfer patients to appropriate stroke facilities for thrombolysis or thrombectomy-capable centers for EVT.

When a blood vessel is blocked in ischemic stroke, the primary blood supply to a circumscribed area of the brain is compromised. This leads to a reduced supply of oxygen and glucose and thus depletes energy/adenosine triphosphate to that brain area, as well as increasing metabolic end products such as carbon dioxide and lactate. This results in an acidotic shift (17–19). Evolving ischemia and reduced cellular energy impair metabolic pumps, damage cells, and activate the innate immune system in the microvessels and brain overall (20). Local neurovascular inflammation is largely driven by inflammatory signaling, inflammatory molecules such as metalloproteinases, and cytokines such as tumor necrosis factor-alpha (TNF-alpha). This activates immune cells and damages the extracellular matrix and the blood–brain barrier, resulting in the entry of toxic blood constituents including activated leukocytes (20–23). Neurovascular inflammatory processes involve interactions among E-Selectin, P-Selectin, and L-Selectin (i.e., small protein molecules expressed on leukocytes/endothelium) that mediate the first contact between stimulated endothelial cells and leukocytes. Selectins are involved in initial interactions/rolling of leukocytes. P-selectin facilitates the rolling of platelets and leukocytes on activated endothelial cells. Upon platelet activation, P-selectin translocates from intracellular granules to the outer membrane, while fibrinogen aggregates platelets by binding glycoprotein (GP) IIb/IIIa between adjacent platelets. Up-regulated integrin and Ig superfamily cell adhesion molecules provide firm adhesion and their subsequent extravasation into the brain from the vascular endothelium to initiate immune cell homing, platelet binding, and neutrophil extravasation. Thus, Vascular Cell Adhesion Molecule-1 (VCAM-1) and Intercellular Adhesion Molecule-1 (ICAM-1) are responsible for cell adhesion of circulating immune cells enabling leukocyte migration from the leaky vessels into the brain (24, 25). The evolution of brain injury continues to increase with migration of leukocytes, glial activation and inflammation, and accumulation of necrotic and apoptotic cells (i.e., increased cell death and an expanding brain infarction) (24).

In AIS, these pathophysiological processes lead to cell damage and death and cause the release of cell proteins inside and on the surface of the brain and vascular cells into the bloodstream. Glial fibrillary acid protein (GFAP), which is found in astrocytes, is responsible for the stability of the intracellular structure (26). Neuron-specific enolase (NSE) is involved in the energy metabolism of neurons via catalysis of 2-phosphoglycerate to phosphoenolpyruvate (27). The S100 protein family consists of at least 20 different, multifunctional signaling proteins involved in regulating various cellular processes such as motility, differentiation, cell cycle progression, and cell growth (28). One member of the S100 family, S100B, is found in astrocytes, and oligodendrocytes. These proteins are released into the blood when cells are injured by a disruption of the blood–brain barrier, as in AIS (29).

Vascular occlusion in AIS not only leads to brain insults but also has consequences on the autonomic nervous and cardiovascular systems (30). The interaction between brain and heart is often referred to as stroke-heart syndrome, which is associated with a vegetative derailment that primarily affects blood pressure and heart rate, but can also include cardiac arrhythmias or cardiac shock (31). Proteins such as N-Terminal pro-Brain Natriuretic Peptide (NT-proBNP) or Brain Natriuretic Peptide (BNP) are increasingly released, when the heart is being stressed, as in AIS (32–34).

In this systematic review, we summarize the literature on blood-based protein biomarkers into the following pathophysiological classes: neurovascular inflammation (MMP-9, TNF-alpha), endothelial function (VCAM-1, ICAM-1), cell migration (E-selectin, P-selectin, L-selectin), markers of glial, astrocytic neuronal origin (NSE, GFAP, S100, S100B) and cardiac dysfunction (BNP, NT-proBNP). These biomarkers play key roles in the progressive ischemic brain injury and pathophysiology that occurs after AIS. Our review includes studies reporting blood-based biomarker levels measured within 24 h after the onset of AIS symptoms to provide an up-to-date overview of individual potential blood-based protein biomarkers that may aid acute AIS diagnosis. A secondary objective was to screen and analyze included studies that measured more than one of these biomarkers in AIS patients and to summarize these results.

## 2 Materials and methods

### 2.1 Study criteria

The clinical research question (i.e., Population, Intervention, Controls, Outcome, and Time; PICOT) and the following inclusion criteria were: (1) Population: patients with AIS, age ≥ 18 years; (2) Intervention: measurement of blood MMP-9, TNF-alpha, VCAM-1, ICAM-1, E-Selectin, P-Selectin, L-Selectin, NSE, GFAP, S100, S100B, BNP and NT-proBNP levels; (3) Inclusion of a control group: healthy controls (HC), matched controls, and/or stroke mimics as indicated by the authors of the included studies (i.e., syncope, migraine, neuropathy, brain tumor, toxic-metabolic disturbances, systemic infections, postictal state, headache disorders, vestibular disorders, seizure, symptomatic aggravation of known neurodegenerative disorders, peripheral neuropathy, syncope, brain tumor, metabolic disorders, functional disorders, infections, transient global amnesia, hearing loss, etc.); (4) Outcomes included comparison of biomarker levels between AIS patients and controls; (5) Time interval: studies up to November 2023; (6) Study design: randomized controlled trials, prospective cohort studies, case–control studies, retrospective studies; and (7) English language.

Exclusion criteria were: (1) Published whole text language other than English; (2) Missing control group; (3) Animal studies; and (4) Case reports, case series, reviews, and letters to the editor.

### 2.2 Data sources and search strategy

This systematic review followed the “Preferred Reporting Items for Systematic Reviews and Meta-Analyses” (PRISMA) guidelines (35). The literature search was performed by two independent investigators (JR, AC) using EMBASE, The Web of Science, The Cochrane Library, and MEDLINE up to November 2023.

Combinations of search strings included “stroke,” “protein,” “biomarker,” “NSE,” neuron specific enolase,” “S100,” “S100B,” “GFAP,” “glial fibrillary acid protein,” MMP-9″, “metalloproteinase,” “ICAM-1″,” intercellular adhesion molecule,” “VCAM-1″, “vascular cell adhesion molecule,” “Selectin,” “BNP,” “B-type natriuretic peptide,” “NT-proBNP,” “TNF-alpha,” “tumor necrosis factor” with the Boolean operators “AND” and “OR.” The Boolean operator “NOT” was used for the terms, “mice,” “mouse,” “rat,” “rats,” and “animal.” (36) Reference lists of identified articles were screened for additional sources. Furthermore, relevant meta-analyses and systematic reviews were screened manually to ensure a comprehensive literature review. The complete search strategy can be found in the “Appendix_Literature_Search_Strategy.” Zotero was used to screen literature search results and remove duplicates (37).

### 2.3 Data extraction

The literature review was performed by two independent investigators (JR, AC). A third reviewer (FCB) was involved in case of disagreement between the two primary reviewers. The following data were extracted from the publications: (1) study characteristics (type of study, time of investigation, number of included AIS and controls); (2) participants’ baseline characteristics (age, sex/gender); (3) biomarker measurement methods used, units of measurements; (4) blood drawing time (range); (5) study outcome (biomarker comparisons between AIS patients and control groups. If outcome-related data could not be obtained by searching the articles for text, manuscript tables, and supplemental tables, data were extracted using the WebPlotDigitizer if an adequate graphic was provided) (38). If not otherwise specified, significance levels of the included studies were *p*-value of ≤0.05. All results reported in this review were taken from the original articles and are presented according to their specifications.

### 2.4 Methodological quality assessment—risk of bias

The quality of case–control and cohort studies was evaluated according to the “Newcastle-Ottawa Scale” (NOS). The developers of the NOS established a “star system” in which studies are rated in the categories “Selection, Comparability, and Exposure/Outcome.” (39) A maximum of 9 points can be achieved. In line with the current literature the studies were classified as follows: ≥7 stars were considered as “good-quality,” 2–6 stars, “fair-quality,” and ≤ 1 star, “poor-quality.”

## 3 Results

**Figure 1:**
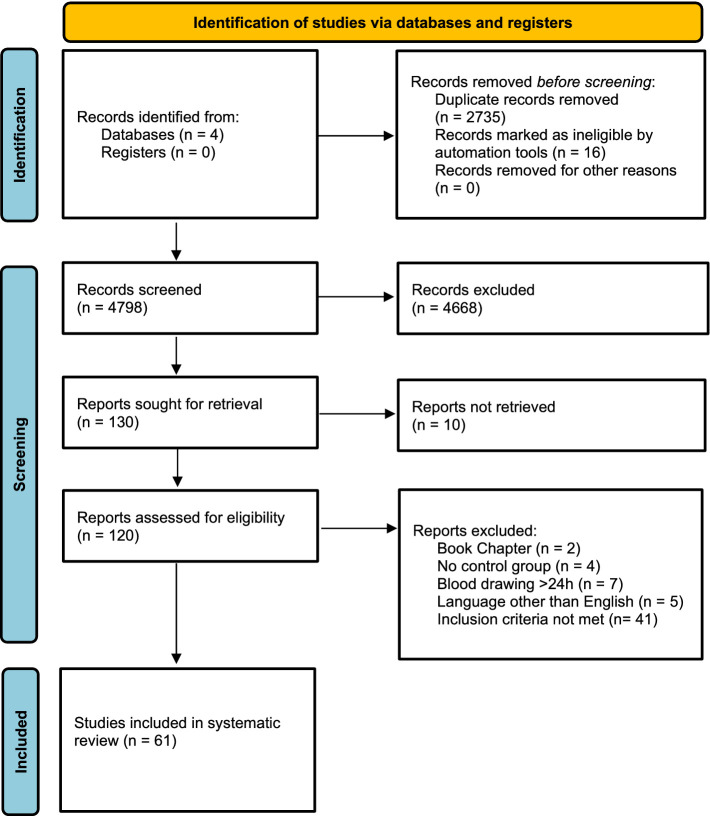
PRISM flow diagram.

The initial search used EMBASE, The Web of Science, The Cochrane Library, and MEDLINE and resulted in 7,533 publications. After removing duplicates, 4,798 publications were screened. Of those, 130 were eligible for a full-text search. Subsequently, 69 were excluded, 10 could not be retrieved, two were book chapters, four did not include a control group, five were written in a language other than English, and 48 did not meet our inclusion criteria (e.g., investigation of other biomarkers than stated in the inclusion criteria, blood sampling >24 h after symptom onset or the timing of blood sampling was uncertain) (Figure 1). Considering that individual studies sometimes investigated more than one biomarker, we were able to detect a total of 61 unique studies that reported, in total, on 4,644 AIS patients and 2,777 participants in a control group. The number of studies reporting on each of the biomarkers is as follows: MMP-9, TNF-alpha, *n* = 10; *n* = 20; VCAM-1, *n* = 9; ICAM-1, *n* = 10, E-Selectin, *n* = 5, P-Selectin, *n* = 3, L-Selectin, *n* = 2, NSE, *n* = 10, GFAP, *n* = 11, S100, *n* = 5, S100B, *n* = 9, BNP, *n* = 7, NT-proBNP *n* = 3, biomarker panels, *n* = 8. Most studies were carried out in Italy (14.8%), Germany (11.5%), and Turkey (11.5%). Interestingly, 20 different countries were represented in the included studies (Table 1; Supplementary Figure S1). Overall, the NOS bias assessment showed that most of the studies evidenced a fair quality (70.5%). Good quality was present in 29.5%. Poor quality was not detected. On average, a value of 5.36 (standard deviation, SD: 1.752) stars was observed (Supplementary Tables S1–S5).

**Table 1:** Study overview by blood-based biomarker for AIS acute ischemic stroke.

Category	Biomarker	Publication year	Country	Studies N	AIS N	Control N	Controls	Blood drawing time (range)	Outcome AIS vs. controls, *p* < 0.05
Inflammation	MMP-9	2001–2023	Spain (*n* = 5), USA (*n* = 4), South Korea (*n* = 3), Italy (*n* = 2), Croatia (*n* = 1), Turkey (*n* = 1), China (*n* = 1), Germany (*n* = 1), Egypt (*n* = 1), Brazil (*n* = 1)	15	902	931	Healthy controls, matched controls	20 min to 24 h	6/15
				5	1,603	355	Stroke mimics		3/5
	TNF-alpha	2001–2023	Italy (*n* = 5), Finland (*n* = 1), USA (*n* = 1), Poland (*n* = 1), South Korea (*n* = 1), Brazil (*n* = 1)	8	546	532	Healthy controls, matched controls, no specification (*n* = 1)	7–24 h	5/8
				2	196	97	Stroke mimics		0/2
Endothelial integrity	VCAM-1	1995–2013	Italy (*n* = 4), Croatia (*n* = 2), Germany (*n* = 1), Greece (*n* = 1), United Arab Emirates (*n* = 1)	9	807	645	Healthy controls, matched controls	4–24 h	5/9
	ICAM-1	1995–2013	Italy (*n* = 4), Croatia (*n* = 2), Germany (*n* = 1), United Arab Emirates (*n* = 1), Greece (*n* = 1), Taiwan (*n* = 1)	10	858	670	Healthy controls, matched controls	4–24 h	6/10
Cell migration	E-Selectin	1997–2010	Italy (*n* = 2), Croatia (*n* = 1), United Arab Emirates (*n* = 1), Taiwan (*n* = 1)	5	385	368	Healthy controls, matched controls, hospitalized non-stroke patients	<12–24 h	3/5
	P-Selectin	2003–2009	Italy (*n* = 2), South Korea (*n* = 1)	3	252	249	Matched controls, hospitalized non-stroke patients	<12–24 h	3/3
	L-Selectin	1995–2004	Germany (*n* = 1), Croatia (*n* = 1)	2	89	99	Matched controls	4–24 h	1/2
Glial and neuronal origin	NSE	1991–2018	Germany (*n* = 2), South Korea (*n* = 2), Turkey (*n* = 1), USA (*n* = 1), Ireland (*n* = 1), Netherlands (*n* = 1), Spain (*n* = 1), China (*n* = 1)	7	316	329	Healthy controls, matched controls	4–24 h	4/7
				3	615	198	Stroke mimics		1/3
	GFAP	2012–2023	Germany (*n* = 3), China (*n* = 1), Greece (*n* = 1), Norway (*n* = 1), India (*n* = 1), USA (*n* = 1), Italy (*n* = 1), South Korea (*n* = 1), Turkey (*n* = 1)	6	424	342	Healthy controls	1.9–24 h	3/6
				7	728	275	Stroke mimics		1/7
	S100	1997–2011	Germany (*n* = 3), China (*n* = 1), Turkey (*n* = 1)	5	302	184	Healthy controls, matched controls	4–24 h	3/5
	S100B	2003–2017	South Korea (*n* = 3), Spain (*n* = 2), USA (*n* = 2), Italy (*n* = 1), Turkey (*n* = 1)	2	127	271	Healthy controls	3.5–24 h	1/2
				8	2,107	937	Stroke mimics		5/8
Cardiac dysfunction	BNP	2004–2016	Turkey (*n* = 2), South Korea (*n* = 1), Italy (*n* = 1), USA (*n* = 1), Japan (*n* = 1), Spain (*n* = 1)	4	277	186	Healthy controls	3.5–24 h	4/4
				3	1,036	187	Stroke mimics		2/3
	NT-proBNP	2005–2017	Greece (*n* = 1), Turkey (*n* = 1), Spain (*n* = 1)	2	87	87	Healthy controls	<6–24 h	2/2
				1	941	193	Stroke mimics		0/1

N, number of patients; AIS, acute ischemic stroke.

### 3.1 Biomarkers of neurovascular inflammation

#### 3.1.1 MMP-9

Twenty studies in which MMP-9 was measured were included and comprised 2,515 AIS patients and 1,286 controls (HC/matched controls: *n* = 931, stroke mimics: *n* = 355) (22, 34, 40–57). Most investigations occurred in Spain (*n* = 5) followed by the USA (*n* = 4), South Korea (*n* = 3), Italy (*n* = 2), Croatia (*n* = 1), Turkey (*n* = 1), China (*n* = 1), Germany (*n* = 1), Egypt (*n* = 1), and Brazil (*n* = 1). ELISA was the measurement platform in 76%. However, POCT systems as well as heat shock protein pull-down assays and antibody-based arrays other than ELISA were used. Studies were published between 2001 and 2023. Blood drawing time among studies ranged from 20 min to <24 h after symptom onset (Table 2). Seventy percent of the included studies showed good quality according to the NOS, while 30% of the studies were of fair quality (Supplementary Table S1).

**Table 2:** Inflammation biomarkers.

Author	Study type	Study year	Study period	Origin of the study	Blood drawing median [IQR] mean (SD) in h	Measurement method	AIS (N)	AIS age (years), median [IQR] mean (SD)	AIS female/male (N)	Definition controls	Control group (N)	Control age (years), median [IQR] mean (SD)	Control female/male (N)	Outcome (AIS vs. control) median [IQR] mean (SD)
MMP-9—controls other than stroke mimics														
Montaner et al.	Prospective case–control	2001	06/1999–03/2000	Spain	<12	ELISA	39	73.97 (15.34)	19/20	HC	62	43 (n.a.)	26/36	AIS: 147.1 ng/mL (SEM:118.6) vs. HC: 41.0 ng/mL (SEM:27.8), p = n.a. *Unoccluded MCA:* 99.4 ng/mL (102.4) *Proximal occlusion:* 236.4 ng/mL (81.1) *Distal occlusion*: 188.1 ng/mL (149.3)
Castellanos et al.	Prospective, observational	2003	03/1999–02/2000	Spain	4–13.2	ELISA	212	72.2 (8.3)	98/114	HC	34	59 (13)	15/19	AIS with Hemorrhagic Transformation (Htr): 193.0 ng/mL [163–213] vs. AIS without Htr: 62.0 ng/mL [40–93], *p* < 0.001 AIS with Htr vs. HC: 56 ng/mL [39–79], *p* < 0.001 *LAAS*: AIS + Htr: 213 ng/mL [137–217], AIS without Htr: 50.0 ng/mL [3–99], p = 0.002
Castellanos et al.	Prospective, observational	2003	03/1999–02/2000	Spain	4–13.2	ELISA	212	72.2 (8.3)	98/114	HC	34	59 (13)	15/19	*CEI*: AIS + Htr: 193 ng/mL [169–212] vs. AIS without Htr: 64 ng/mL [43–88], *p* < 0.001 *ODE*: AIS + Htr: 165 ng/mL [93–217] vs. AIS without Htr: 68 ng/mL [50–124], *p* = 0.025
Heo et al.	Prospective case–control	2003	n.a.	South Korea	0.3–4.5	ELISA	23	66 [n.a.]	11/12	HC	47	46 [n.a.]	24/23	*Nonrecanalization group:* 311.6 ng/mL [173.2–372.3] vs. *Recanalization group:* 122.6 ng/mL [82.4–181.0] vs. HC: 54.2 ng/mL [28.2–98], Kruskal-Wallis: *p* < 0.001
Horstmann et al.	Prospective case–control	2003	n.a.	Germany	<12	ELISA	50	60 (n.a.)	17/33	HC	50	59 (n.a.)	17/33	AIS: 26,674.00 integrated density (7428.79) vs. HC: 5221.0 integrated density (5349.36), *p* ≤ 0.05
Montaner et al.	Prospective case–control	2003	n.a.	Spain	<3	ELISA	41	70 (10.6)	25/16	HC	62	43 (n.a.)	26/36	AIS: 135.4 ng/mL (122.8) vs. HC: 41 ng/mL (27.8), p = n.a.
Reynolds et al.	Prospective case–control	2003	11/1999–02/2003	USA	12	ELISA	38	n.a.	n.a.	HC	214	n.a.	n.a.	AIS: 262 ng/mL [61.54–779.49] vs. HC: 34.62 ng/mL [21.80–65.38], p = n.a. OR: 4.21 (95% CI: 2.57–8.02)
Ning et al.	Prospective case–control	2006	02/2002–02/2004	USA	<8	ELISA	26	tPA-treated AIS: 69.9 (13.1)	11/15	Age- and sex-comparable subjects without a clinical history of stroke	27	68.9 (10.6)	25/12	AIS: 46 ng/mL [28–124] vs. controls: 28.6 ng/mL [16.05–51.47], *p* > 0.05
							26	tPA-untreated AIS: 72 (15)	17/9					AIS: 28 ng/mL [16;33] vs. controls: 28.6 ng/mL [16.05–51.47], *p* > 0.05
Vukasovic et al.	Prospective case–control	2006	08/2002–07/2003	Croatia	<24	ELISA	126	70 (9)	58/68	HC recruited from the Zagreb-Zapad Health Center, Zagreb	124	68 (8)	83/41	*All AIS*: 485.0 ng/mL (250) vs. *HC:* 436 ng/mL (184), *p* > 0.05 *LAC*: 420.0 ng/mL (247), *Partial anterior infarction*: 486.0 ng/mL (256); *Total anterior infarction:* 596 ng/mL (240); *Posterior circulation infarction:*
														339.0 ng/mL (162), Anova *p* ≤ 0.05 *ROC-analysis:* Total anterior occlusion: Cut-off: 671 ng/mL (sensitivity: 0.457, specificity: 0.857); OR: 5.053 (95% CI: 2.081–12.269), *p* < 0.001
Lucivero et al.	Prospective case–control	2007	04/2004–10/2004	Italy	<12	ELISA	29	72 (.)	20/9	HC	37	64 (n.a.)	18/19	*All AIS:* 423.9 ng/mL (185.3) vs. *HC*: 375.4 ng/mL (176.0), *p* > 0.05; Partial anterior AIS: 409.8 ng/mL (192.2); LAC: 453.6 ng/mL (177.2) vs. HC, *p* > 0.05
Kim et al.	Prospective case–control	2010	04/2007–08/2007	South Korea	6	Triage^®^ Meter POCT instrument (Biosite, Inc.).	89	66.6 (11.8)	39/50	HC	57	43.8 (12)	32/25	AIS: 242.1 ng/mL (242.6) vs. HC: 211.2 ng/mL (184.8), p = n.a.
Demir et al.	Prospective case–control	2012	n.a.	Turkey	<6	ELISA	32	66.9 (11.2)	16/16	Age and gender-matched HC	30	63.8 (8.9)	15/15	*Baseline*: 9.611 ng/mL (5.726) vs. 5.942 ng/mL (3.011), *p* = 0.003 *12th h*: 9.12 ng/mL (8.506) vs. 5.942 ng/mL (3.011), *p* = 0.054 *24th h:* 10.055 ng/mL (7.850) vs. 5.942 ng/mL (3.011), *p* = 0.009
Lehmann et al.	Prospective case–control	2015	01/2012–09/2014	Brazil	<24	ELISA	35 (LAAS)	68,5 [63.5–74]	13/22	Age, ethnicity, and body mass index controlled HC from the Blood Bank of Londrina and from general population of Londrina	96	69	73	AIS: 1137.0 ng/mL [809.0;1813.0] vs. controls: 686.4 ng/mL [544.8–1048.0], *p* < 0.01
							39 (LAC)	73 [61–78]	18/21					AIS: 1367.0 ng/mL [763.5–1775.0] vs. controls: 686.4 ng/mL [544.8–1048.0], *p* < 0.01
							21 (CEI)	68,5 [63.5–74]	8/13					AIS: 1307.0 ng/mL [814.5–1750.0] vs. controls: 686.4 ng/mL [544.8–1048.0], *p* ≤ 0.05
Abdelnaseer et al.	Prospective case–control	2017	n.a.	Egypt	<24	ELISA	30	61 (7.11)	15/15	Matched age and sex HC	30	63 (6.78)	16/14	AIS: 998.8 ng/mL (154.72) vs. HC: 691.8 ng/mL (232.37), *p* = 0.03
Li et al.	Prospective case–control	2022	01/2019–09/2019	China	<24	ELISA	42	56.81 (10.81)	20/22	HC	40	57.15 (11.43)	20/20	AIS: 69.69 ng/mL (20.56) vs. HC: 41.52 ng/mL (15.13), *p* < 0.001
Kowalski et al.	Prospective case–control	2023	08/2021–04/2022	USA	24	Extracellular vesicles isolated by HSP pull down	4	65.7 (20.02)	n.a.	2 pooled samples of blood from HC	21	53 (11); 54 (9.5)	11/10	AIS: 4486.5 pixel density [3584.25–5564.5] vs. HC: 1765 pixel density [0], *p* = n.a.
MMP-9—stroke mimics														
Montaner et al.	Prospective, observational	2010	n.a.	Spain	24	ELISA	915	72.63 (12.46)	443/471	Stroke mimics	90	69.57 (17.13)	34/65	AIS: 267 ng/mL [149.42–468.81] vs. stroke mimics: 194.66 ng/mL [107.58–404.42], *p* = 0.019 *ROC-analysis:* MMP-9 > 199 ng/mL: (sensitivity: 0.65; specificity: 0.53); *Logistic regression:* OR = 1.66 (95% CI 1.01–2.73), *p* = 0.046
Glickman et al.	Prospective, observational	2011	n.a.	USA	3.5 (8)	ELISA	34	65.2 (16.2)	22/12	Stroke mimics	29	50.9 (19.1)	20/9	Missing units: AIS: 301.5 (157.5) vs. stroke mimics: 151.9 (74.2), *p* < 0.001; OR 6.83 (2.30–20.31)
Vanni et al.	Prospective, observational	2011	06/2006–09/2006	Italy	6 (7)	Triage^®^ Meter point-of-care instrument	87	74 (11)	34/53	Stroke mimics	68	69 (16)	31/38	AIS: 175 ng/mL (149) vs. stroke mimics: 175 ng/mL (227), *p* = 0.980
An et al.	Prospective, observational	2013	09/2010–10/2010	South Korea	11 [7–16]	ELISA	188	66 (11)	87/101	Stroke mimics	90	61 (9)	53/37	AIS: 63.3 ng/mL [29.7–122.8] vs. stroke mimics: 33.8 ng/mL [15.4–60.8], *p* < 0.001; OR, 1.71 (95% CI: 1.33–2.21; *p* < 0.001)
Bustamante et al.	Prospective, observational, multicenter	2017	08/2012–11/2013	Spain	<6	Antibody-based array (Search Light Technology, Aushon BioSystems, Billerica)	389	n.a.	n.a.	Stroke mimics	78	n.a.	n.a.	AIS: 11.6 pg/mL [11.2–12.3] vs. stroke mimics: 11.8 pg/mL [11.3–12.2], *p* = n.a.
TNF-alpha—controls other than stroke mimics														
Zaremba et al.	Prospective case–control	2001	n.a.	Poland	<24	ELISA	23	72.2 (10.8)	n.a.	Patients with neurasthenia and tension headaches served as a control sex and age-matched	15	n.a.	n.a.	AIS: 14.0 pg/mL (10.2) vs. control: 9.1 pg/mL (1.6), *p* ≤ 0.05
Intiso et al.	Prospective case–control	2004	n.a.	Italy	<24	ELISA	41	67.6 (12.5)	22/19	Age- and sex-matched HC	40	66.4 (9.8)	22/18	AIS: 30.1 pg/mL (12.5) vs. HC: 29 pg/mL (13.9), *p* = 0.746
Sotgiu et al.	Prospective, observational	2006	n.a.	Italy	<20	ELISA	50	68 (n.a.)		Age- and sex-matched in- and out-patients with other neurological diseases	32	62 (.)	11/21	AIS: 75 pg/mL [376.2–138] vs. controls: 37.9 pg/mL [36.1–47.2], *p* = 0.001
Licata et al.	Prospective, observational	2009	11/2002–01/2006	Italy	<12	ELISA	120	72 [64–82.5]	52/68	Patients admitted for any cause other than acute cardiovascular/cerebrovascular events	123	69 [65–83]	68/55	AIS: 31.5 pg/mL [10.25–41] vs. controls: 3.7 pg/mL [1.1–4.3], *p* < 0.001 LAAS: 27.5 pg/mL [13.67–40.40] CEI: 38.5 pg/mL [22.2–46] LAC: 19.4 pg/mL [9–23] ODE: 28.9 pg/mL [10.43–38.95]
Tuttolomondo et al.	Prospective, observational	2009	11/2006–10/2008	Italy	<12	ELISA	107	71 [63–80.5]	41/66	Matched for age (±3 years), sex, and cardiovascular risk factor prevalence	102	68 [63–80]	55/57	*All stroke:* 30.5 pg/mL [10.25–46] vs. 5.1 pg/mL [1.1–4.3], *p* < 0.001; *LAAS*: 29.5 pg/mL [15.3–44.5] *LAC*: 18.4 pg/mL [11.0–23.0], *CEI*: 37.2 pg/mL [21.2–48.0] *ODE*: 27 pg/mL [11.4–33.0], *p* < 0.0001
Cevik et al.	Prospective case–control	2013	01/2011–12/2011	Finland	<24	ELISA	60	57.9 (10.2)	23/35	No specification	45	51.05 (9.07)	n.a.	28.07 pg/mL (2.08) vs. 6.07 pg/mL (2.31), *p* < 0.001
Musumeci et al.	Prospective, observational	2013	n.a.	Italy	<24	ELISA	50	68.5 (11.36)	19/31	HC	79	62.54 (7.67)	33/46	NIHSS≤30: 52.74 pg/mL (26.86) vs. NIHSS>30: 225.32 pg/mL (163.60), *p* = 0.0001; Control: 0.0–13.3 pg/mL
Lehmann et al.	Prospective case–control	2015	01/2012–09/2014	Brazil	<24	ELISA	35 (LAAS)	68,5 [63.5–74]	13/22	Age, ethnicity, and body mass index controlled HC from the Blood Bank of Londrina and from the general population of Londrina	96	69	73	AIS: 2 pg/mL [2.0–3.39] vs. HC: 2.0 pg/mL [2.0–6.85], *p* > 0.05
							39 (LAC)	73 [61;78]	18/21					AIS: 2.25 pg/mL [2.0–5.27] vs. HC: 2.0 pg/mL [2.0–6.85], *p* > 0.05
							21 (CEI)	68,5 [63.5–74]	8/13					AIS: 2.0 pg/mL [2.0–5.03] vs. HC: 2.0 pg/mL [2.0–6.85], *p* > 0.05
TNF-alpha—stroke mimics														
An et al.	Prospective, observational	2013	09/2010–10/2010	South Korea	11 [7–16]	ELISA	188	66 (11)	87/101	Stroke mimics	90	61 (9)	53/37	AIS: 2.2 pg/mL [0.0;8.9] vs. stroke mimic: 2.6 pg/mL [0.0–7.4], *p* = 0.541
Kowalski et al.	Prospective case–control	2023	08/2021–04/2022	USA	24	ELISA	8	n.a.	n.a.	Stroke mimics	7	53 (11); 54 (9.5)	11/10	AIS: 0.47 pg/mL [0.34–0.74] vs. stroke mimic: 0.55 [0.41–5.70], *p* = n.a.

LAAS, Large Artery Atherosclerosis Stroke; LAC, Lacunar infarct; CEI, Cardio-Embolic Infarct; LVO, Large vessel occlusive stroke; SVD, Small Vessel Disease; Afib, Atrial fibrillation; TIA, Transient Ischemic Attack; ODE, Other Determined Etiology; AIS, Acute Ischemic Stroke; h, hours; N, Number of patients; SD, Standard deviation; SEM, Standard Error of the Mean; IQR, Inter Quartile Range; n.a., not applicable; AUC, Area Under Curve; ROC, Receiver Operating Characteristic; OR, Odds Ratio; HC, Healthy Controls; ELISA, Enzyme-linked Immunosorbent Assay; Elecsys, ElectroChemiLuminescence ImmunoAssay System; Eclia, ElectroChemiLuminescence ImmunoAssay; FacScan, Fluorescence Activated Cell Scanner; SIMOA, Single Molecule Array; HSP, Heat Shock Proteins; tPA, tissue Plasminogen Activator; TNF, Tumor Necrosis Factor; MMP-9, Matrix metalloproteinase 9.

##### 3.1.1.1 AIS vs. healthy controls/matched controls

Most of the studies measuring MMP-9 (*n* = 15; 80%) compared AIS patients to HC/matched controls. Higher MMP-9 levels in AIS patients compared to HC were observed generally in 47% of studies (22, 43–45, 52, 54, 56) (Table 1). In 20% of studies there was a significant difference (*p* < 0.05) (47–49). No statistical comparison was made between AIS and HC in 33% of the studies, yet 80% of those studies reported at least twice as high MMP-9 levels in AIS patients compared to HC (34, 40, 41, 44, 46, 57).

##### 3.1.1.2 AIS vs. stroke mimics

Patients with stroke mimics as a control group were observed in 20% of studies. Three of them showed higher MMP-9 levels in AIS patients compared to stroke mimics (*p* ≤ 0.05), although two studies did not perform statistical comparisons between AIS and stroke mimic patients (Table 1) (42, 51, 53). In two studies, almost similar MMP-9 levels were observed in AIS and stroke mimic patients (175 ng/mL ± 149 vs. 175 ng/mL ± 227, *p* = 0.980; and median 11.6 pg/mL, IQR: 11.2–12.3 vs. 11.8 pg/mL, IQR: 11.3–12.2) (50, 55). In a receiver operator curve (ROC) analysis to differentiate between AIS and stroke mimics based on MMP-9 levels, a cut-off value of 199 ng/mL showed a sensitivity of 65% and a specificity of 53%. The odds ratio (OR) of AIS versus stroke mimic was 1.66 (95% CI: 1.01–2.73, *p* = 0.046) (42). In another study, analyzing MMP-9 using a bootstrap method there was moderate predictive power for AIS, ranging from 42.4% (backward selection) to 75.5% (forward selection).

##### 3.1.1.3 Etiology-, outcome-, severity related MMP-9 levels in AIS patients

Compared to HC, higher MMP-9 levels were reported in one study investigating patients experiencing large artery atherosclerosis strokes (LAAS) 1137.0 ng/mL [IQR: 809.0–1813.0], lacunar stroke (LAC) 1367.0 ng/mL [IQR: 763.5–1775.0], and cardioembolic infarcts (CEI) 1307.0 ng/mL [IQR: 814.5–1750.0] vs. 686.4 ng/mL [IQR: 544.8–1048.0]; (*p* ≤ 0.05) (54). Studying MMP-9 levels in patients with unoccluded middle cerebral artery (MCA), proximal occlusion of the MCA, or distal occlusion of the MCA using transcranial Doppler revealed the more distal the pathology, the higher the MMP-9 levels (unoccluded MCA: 99.4 ng/mL, SD: 102.4; proximal occlusion: 236.4 ng/mL ± 71.1; distal occlusion: 188.1 ng/mL ± 149.3, Kruskal-Wallis-test *p* = 0.032) (40). Moderate to high correlations between infarct volume measured by brain CT or MRI on admission and MMP-9 concentrations were reported (*r* = 0.35–0.89, *p* < 0.001, respectively) (22, 43, 45, 47, 52). However, two studies reported correlations *p* > 0.05, albeit early signs of ischemia on brain CT were associated with higher levels of MMP-9 (163 ng/mL [IQR: 110–193] vs. 54 ng/mL [IQR: 38–74], *p* < 0.001) (40, 43). MMP-9 concentrations have also correlated positively with the severity of stroke as determined by the National Institutes of Health and Stroke Scale (NIHSS) after 12 h (*r* = 0.46, *p* = 0.01), but not with the NIHSS measured on admission (*r* = 0.28, *p* = 0.12) (52). Interestingly, mean MMP-9 levels did not differ in the presence versus absence of hypertension (118.3 ng/mL versus 104.1 ng/mL, *p* = 0.58) (41). However, one study reported a positive correlation between plasma MMP-9 levels and both systolic (*r* = 0.70) and diastolic blood pressure (*r* = 0.63, *p* ≤ 0.05) (43).

#### 3.1.2 TNF-alpha

Ten eligible studies measuring TNF-alpha were found comprising 742 AIS patients and 629 controls (HC/matched controls: *n* = 532 stroke mimics: *n* = 97) (53, 54, 57–64). Half of the studies were carried out in Italy (*n* = 5) followed by Finland (*n* = 1), USA (*n* = 1), Poland (*n* = 1), South Korea (*n* = 1), and Brazil (*n* = 1). Studies were published between 2001 and 2023 and in all studies, ELISA was used to measure TNF-alpha. Only two studies used stroke mimics as a control group while the others included age-, and sex-matched healthy controls or patients admitted to the hospital for a cause other than acute cardiovascular or cerebrovascular events. Blood collection among studies ranged from 7 h to <24 h after symptom onset (Table 2). The quality of the studies was found to be good in 50% of the studies and fair in 50% of the included studies (Supplementary Table S1).

##### 3.1.2.1 AIS vs. healthy controls/matched control patients

More than half of the included studies (62.5%) showed higher levels of TNF-alpha in AIS patients compared to the control group, 25% revealed lower TNF-alpha levels and one study did not provide a statistical comparison (58, 60–64).

##### 3.1.2.2 AIS vs. stroke mimics

One study revealed no difference in TNF-alpha levels between AIS patients and stroke mimics (2.2 pg/mL, IQR: 0–8.9 vs. 2.6 pg/mL, IQR: 0–7.4, *p* = 0.541) (53). Comparable results were obtained by another study (0.47 pg/mL, IQR: 0.34–0.74 vs. 0.55 pg/mL, IQR: 0.41–5.70) (57).

##### 3.1.2.3 Etiology-, outcome-, severity related TNF-levels in AIS patients

Among AIS classified as CEI, compared to LAAS, LAC, or other determined etiology (ODE), higher median TNF-alpha plasma levels were reported (*p* < 0.0001) (61, 62). Contrary results were reported by one study that did not find differences by AIS etiology (LAAS, LAC, CEI) and age-, ethnicity-, and body mass index-matched healthy controls (54). No differences in TNF-alpha levels were found in another study (30.1 pg/mL, SD:12.5 vs. 29.0 pg/mL, SD: 13.9, *p* = 0.746) (59). Among patients with LAC and without a lacunar detectable lesion on neuroimaging, no difference in TNF-alpha levels was found (*p* = 0.87) (61). Severely affected stroke patients measured via the NIHSS had higher TNF-alpha levels compared to less severely affected patients (NIHSS>30; 225.32 pg/mL, SD: 163.6 vs. NIHSS ≤30 52.744 pg/mL, SD: 26.86, *p* = 0.0001) (64). A multivariate analysis showed that gender, age, vascular risk factors and the occurrence of inflammatory complications had no influence on TNF-α levels (59).

### 3.2 Biomarkers of endothelial integrity

#### 3.2.1 VCAM-1

Nine studies measured VCAM-1 comprised of 807 AIS patients and 645 controls (60–62, 64–69). Most studies were carried out in Italy (*n* = 4, 44%), followed by Croatia (*n* = 2), Germany (*n* = 1), Greece (*n* = 1), and United Arab Emirates (*n* = 1). Year of publication ranged between 1995 and 2013 and ELISA was the measurement of choice. Blood collection time was reported with a range of 4 h to <24 h after stroke onset (Table 3). Five of the studies showed a fair quality while 4 revealed a good quality. Poor quality was not observed (Supplementary Table S2).

**Table 3:** Endothelial integrity biomarkers.

Author	Study type	Study year	Study period	Origin of the study	Blood drawing median [IQR] mean (SD) in h	Measurement method	AIS (N)	AIS age (years), median [IQR] mean (SD)	AIS female/male (N)	Definition controls	Control Group (N)	Control age (years) median [IQR] mean (SD)	Control female/male (N)	Outcome (AIS vs. control) median [IQR] mean (SD)
VCAM-1														
Fassbender et al.	Prospective case–control	1995	n.a.	Germany	4, 8, 10, 24	ELISA	22	72 (n.a.)	14/8	HC/Vascular risk factor (VRF)	22	69 (n.a.)	14/8	At 4 h: AIS: 1038.5 ng/mL (SEM:88.26) vs. *HC*: 732 ng/mL (SEM: 52), *p* ≤ 0.05 AIS vs. *VRF*: 722.0(SEM: 56.57), *p* < 0.05
Simundic et al.	Prospective case–control	2004	n.a.	Croatia	<24	ELISA	67	72 (n.a.)	30/37	Controls were recruited from the CENTAR Health Center, Zagreb	76	52 (16)	52/24	AIS: 698 ng/mL (289) vs. controls: 660 ng/mL [range: 225–2205], *p* = 0.034, OR: 1.002 (CI: 95% 1.00–1.004), *p* = 0.032
Sotgiu et al.	Prospective, observational	2006	n.a.	Italy	<20	ELISA	50	68 (n.a.)	n.a.	Age-, sex-matched patients with other neurological diseases	32	62 (n.a.)	11/21	AIS: 512.0 ng/mL [414.0–694.0] vs. controls: 419.8 ng/mL [318.3–511.0], *p* = 0.01
Licata et al.	Prospective, observational	2009	11/2002–01/2006	Italy	<12	ELISA	120	72 [64–82.5]	52/68	Patients admitted for any cause other than acute cardiovascular/cerebrovascular events	123	69 [65–83]	68/55	AIS: 20 ng/mL [15.1–23.0] vs. controls: 14 ng/mL [13.0–17.0], *p* < 0.001
Rallidis et al.	Prospective case–control	2009	01/2002–12/2005	Greece	<12	Quantitative sandwich enzyme immunoassay (R & D Systems Europe Ltd., Abingdon, U.K.)	241	54.4 (8)	n.a.	Subjects without evidence of cardiovascular disease, matched for age and sex	76	n.a.	n.a.	AIS: 588 ng/mL [524–655] vs. controls: 576 ng/mL [495–699], *p* > 0.05
Tuttolomondo et al.	Prospective, observational	2009	11/2006–10/2008	Italy	<12	ELISA	107	71 [63–80.5]	41/66	Matched for age (±3 years), sex, and cardiovascular risk factor prevalence	102	68 [63–80]	55/57	*All stroke:* 16 ng/mL [10.1–20.0] vs. controls: 10 ng/mL [7.0–15.0], *p* < 0.001; *LAAS*: 21.0 pg/mL [13.0–22.0], *LAC*: 16.0 pg/mL [20.0–15.0], *CEI*: 20.0 pg/mL [14.7–24.0], *ODE*:16.5 pg/mL [13.0–18.0], *p* = 0.52
Abdulle et al.	Prospective, observational	2010	–	United Arab Emirates	<24	ELISA	40	50.2 (9.5)	4/36	Hospitalized non-stroke patients	42	44.3 (14.9)	9/33	AIS: 232 ng [101–360] vs. controls: 228 ng/mL [55–368], *p* = 0.637
Supanc et al.	Prospective, observational	2011	12/2008–11/2009	Croatia	<24	Quantitative enzyme immunoassay (RandD Systems)	110	70.2 (9.6)	48/62	Examinees of the same age and sex who suffered from headaches and/or dizziness.	93	70 (9.5)	41/52	AIS: 717.5 ng/mL [90–1810] vs. controls: 688 ng/mL [555–850], *p* > 0.05 *LAC*: median: 675 ng/mL, min: 445 ng/mL, max: 1210 ng/mL; *thromboembolic stroke:* median: 730 ng/mL, min: 90 ng/mL, max: 1810 ng/mL, LAC vs. thromboembolic stroke, *p* = 0.023
Musumeci et al.	Prospective, observational	2013	n.a.	Italy	<24	ELISA	50	68.5 (11.36)	19/31	HC	79	62.54 (7.67)	33/46	NIHSS≤30: 519.0 ng/mL (205) vs. NIHSS>30: 683.2 ng/mL (404.0), *p* = 0.064; Control: 15.96 ng/mL (4.024)
ICAM-1														
Fassbender et al.	Prospective case–control	1995	n.a.	Germany	4, 8, 10, 24	ELISA	22	72 (n.a.)	14/8	HC/Vascular risk factor (VRF)	22	69 (n.a.)	14/8	At 4 h: AIS: 420.78 ng/mL (SEM:30) vs. *HC*: 430 ng/mL (SEM: 15), *p* > 0.05 *VRF*: 429.49(SEM: 16.81), vs. no VRF: 310.66 (SEM: 11.04), *p* < 0.001
Shyu et al.	Prospective case–control	1997	n.a.	Taiwan	<24	ELISA	51	66 (12)	21/30	Subjects attending for routine health checkups. symptom-free/medication-free. Venous blood from 10 healthy and young laboratory staff	25	65 (13)	10/15	AIS: 381 ng/mL (SEM: 30) vs. HC: 271 ng/mL (SEM: 27); Young control: 246 ng/mL (SEM: 6), Stroke vs. HC, *p* < 0.01; Stroke vs. Young control, *p* < 0.01
Simundic et al.	Prospective case–control	2004	n.a.	Croatia	<24	ELISA	67	72 (n.a.)	30/37	Controls were recruited from the CENTAR Health Center, Zagreb	76	52 (16)	52/24	AIS: 361 ng/mL (190) vs. controls: 342 ng/mL [range: 180–1188], *p* < 0.001 OR: 1.019 (CI: 95% 1.012–1.026), *p* < 0.001
Sotgiu et al.	Prospective, observational	2006	n.a.	Italy	<20	ELISA	50	68 (n.a.)		Age- and sex-matched in- and out-patients with other neurological diseases	32	62 (n.a.)	11/21	AIS: 274.0 ng/mL [95.0–338.0] vs. controls: 133.5 ng/mL [111.5–183.5], *p* < 0.001
Rallidis et al.	Prospective case–control	2008	01/2002–12/2005	Greece	<12	Quantitative sandwich enzyme immunoassay (R & D Systems Europe Ltd., Abingdon, U.K.)	241	54.4 (8)	n.a.	Subjects without evidence of cardiovascular disease, matched for age and sex	76	n.a.	n.a.	AIS: 267 ng/mL [220–325] vs. controls: 200 ng/mL [179–225], *p* < 0.001
Licata et al.	Prospective, observational	2009	11/2002–01/2006	Italy	<12	ELISA	120	72 [64–82.5]	52/68	Patients admitted for any cause other than acute cardiovascular/cerebrovascular events	123	69 [65–83]	68/55	AIS: 20.8 ng/mL [16.2–24.0] vs. controls: 15.9 ng/mL [12.0–18.1], *p* < 0.001
Tuttolomondo et al.	Prospective, observational	2009	11/2006–10/2008	Italy	<12	ELISA	107	71 [63–80.5]	41/66	Matched for age (±3 years), sex, and cardiovascular risk factor prevalence	102	68 [63–80]	55/57	*All stroke:* 18.8 ng/mL [12.2–20.0] vs. controls: 10.9 ng/mL [12.0–16.1], *p* < 0.001; *LAAS*: 23.8 ng/mL [15.9–24.0], *LAC*: 20.51 ng/mL [15.0–23.0], *CEI*: 22.52 ng/mL [18.55–23.0], *ODE*: 20.25 ng/mL [14.95–18.8], *p* = 0.57
Abdulle et al.	Prospective, observational	2010	n.a.	United Arab Emirates	<24	ELISA	40	50.2 (9.5)	4/36	Hospitalized non-stroke patients	42	44.3 (14.9)	9/33	AIS: 123.2 ng/mL [99.9–134] vs. controls: 118.8 ng/mL [0.0–136], *p* = 0.040
Supanc et al.	Prospective, observational	2011	12/2008–11/2009	Croatia	<24	Quantitative enzyme immunoassay (RandD Systems)	110	70.2 (9.6)	48/62	Examinees of the same age and sex who suffered from headaches and/or dizziness.	93	70 (9.5)	41/52	AIS: 375.55 ng/mL [14.80–745.4] vs. controls: 385.7 ng/mL [213.7–463.9], *p* > 0.05; *LAC*: median: 358.3 ng/mL, min: 14.8 ng/mL, max: 673 ng/mL; *thromboembolic stroke:* median: 376.5 ng/mL, min: 40.5 ng/mL, max: 745.40 ng/mL; LAC vs. thromboembolic stroke, *p* = 0.204
Musumeci et al.	Prospective, observational	2013	n.a.	Italy	<24	ELISA	50	68.5 (11.36)	19/31	HC	79	62.54 (7.67)	33/46	NIHSS≤30: 248.6 ng/mL (86.6) vs. NIHSS>30: 360.35 ng/mL (179.71), *p* = 0.005; Control: 201.2 ng/mL (55.0)

LAAS, Large Artery Atherosclerosis Stroke; LAC, Lacunar infarct; CEI, Cardio-Embolic Infarct; LVO, Large vessel occlusive stroke; SVD, Small Vessel Disease; Afib, Atrial fibrillation; TIA, Transient Ischemic Attack; ODE, Other Determined Etiology; AIS, Acute Ischemic Stroke; h, hours; N, Number of patients; SD, Standard deviation; SEM, Standard Error of the Mean; IQR, Inter Quartile Range; n.a., not applicable; AUC, Area Under Curve; ROC, Receiver Operating Characteristic; OR, Odds Ratio; HC, Healthy Controls; ELISA, Enzyme-linked Immunosorbent Assay; Elecsys, ElectroChemiLuminescence ImmunoAssay System; Eclia, ElectroChemiLuminescence ImmunoAssay; FacScan, Fluorescence Activated Cell Scanner; SIMOA, Single Molecule Array; HSP, Heat Shock Proteins; tPA, tissue Plasminogen Activator; VCAM, Vascular cell adhesion molecule 1; ICAM, Intercellular Adhesion Molecule 1; VRF, Vascular risk factor.

##### 3.2.1.1 AIS vs. healthy controls/matched control patients

Compared to AIS patients, lower VCAM-1 levels were found in 66.67% of control patients (60–62, 65, 66). Contrary results were found in three studies (67–69). The highest measured value (462 ng/mL) was reported in an 86-year-old woman with a severe course of AIS that ended in death (66). Another patient with a high value (1,820 ng/mL) was measured in a 70-year-old man with no history of smoking, myocardial infarction, or hypertension but pre-existing hypercholesterolemia and diabetes mellitus. Surprisingly, his brain CT revealed multiple chronic lacunar ischemic lesions. However, VCAM-1 levels were not associated with age, diabetes mellitus, hypercholesterolemia, hypertension, or degree of arteriosclerosis (66).

##### 3.2.1.2 Etiology-, outcome-, severity related VCAM-1 levels in AIS patients

No differences in median VCAM-1 levels were reported for patients who experienced LAAS, CEI, ODE, or LAC (21 ng/mL, IQR: 13–22; 20 ng/mL, IQR: 14.7–24; 16.5 ng/mL, IQR: 13–18; 16 ng/mL, IQR: 20–15, respectively, Kruskal-Wallis test *p* = 0.52) (62). However, a difference was observed when comparing controls to thromboembolic strokes while no difference was observed for lacunar stroke patients (69). No differences were found in VCAM-1 levels in AIS patients by NIHSS severity (mild, moderate, severe stroke) or outcome measured by the Barthel-Index (69). Nevertheless, higher levels of VCAM-1 were suggested among those with an NIHSS ≥30 compared to those with an NIHSS<30 (683.2 ng/mL, SD: 404 vs. 519 ng/mL, SD: 205, *p* = 0.064) (64). Furthermore, both groups had higher VCAM-1 levels compared to healthy controls (15.96 ng/mL, SD: 4.02) (64). VCAM-1 has been positively correlated with the NIHSS score (*r* = 0.73, *p* < 0.01) and inversely correlated with the Glasgow Coma Scale (GCS) (*r* = −0.64, *p* < 0.01) (60).

#### 3.2.2 ICAM-1

ICAM-1 was measured in 10 studies with a total of 858 AIS patients and 670 controls. There were three studies with healthy controls, four studies with non-stroke patient controls, and three studies with matched controls (60–62, 64–70). Most studies were carried out in Italy (*n* = 4), followed by Croatia (*n* = 2), Germany (*n* = 1), United Arab Emirates (*n* = 1), Greece (*n* = 1), and Taiwan (*n* = 1). Publication years ranged between 1997 and 2013 and the ICAM-1 measurement of choice was ELISA. The blood draw was performed between 4 and 24 h after stroke onset (Table 3). Six of the included studies revealed fair quality and four studies had good quality per the NOS (Supplementary Table S2).

##### 3.2.2.1 AIS vs. healthy controls/matched control patients

Higher ICAM-1 concentrations among patients with AIS compared to the controls were reported in 70% of studies (60–62, 66–68, 70). No difference was found in 20%, while one study did not perform a statistical comparison (64, 65, 69).

##### 3.2.2.2 Etiology-, outcome-, severity related ICAM-1 levels in AIS patients

Four studies did not find differences in ICAM-1 levels by AIS etiology or severity measured using the NIHSS. Nevertheless, one study showed higher ICAM-1 levels in AIS patients who suffered more severe strokes measured by NIHSS (60–62, 64, 69).

ICAM-1 concentrations and involvement of different stroke territories have also been shown to not differ from the control group and no correlations between the extent of brain injury and ICAM-1 levels were observed (61, 70). One study reported that a 10 ng/mL higher ICAM-1 level led to a 9% higher risk of death (60, 64, 67). ICAM-1 levels have been reported to be independent of age, sex, diabetes mellitus, and the degree of atherosclerosis (66). However, one study observed higher ICAM-1 levels in patients with arteriosclerosis (65). In contrast, one study found higher ICAM-1 levels in patients with abnormal carotid ultrasound findings, although the differences were not significant (*p* > 0.05) (70).

### 3.3 Biomarkers of cell migration

#### 3.3.1 E-Selectin

E-Selectin was measured among five studies comprised of 385 stroke patients and 368 controls (61, 62, 66, 68, 70). Eighty percent of studies included patients with conditions other than a neurologic disease, and one study included matched controls (on age, sex, and prevalence of cardiovascular risk factors). Studies were carried out in Italy (*n* = 2), United Arab Emirates (*n* = 1), Croatia (*n* = 1), and Taiwan (*n* = 1). All studies used ELISA for measuring E-Selectin level and dates of publications were between 1997 and 2010. The time of blood sampling after stroke onset was reported as from within <12 h to within <24 h (Table 4). Two studies showed good quality while three studies showed fair quality assessed by the NOS (Supplementary Table S3).

**Table 4:** Cell migration biomarkers.

Author	Study type	Study year	Study period	Origin of the study	Blood drawing median [IQR] mean (SD) in h	Measurement method	AIS (N)	AIS age, median [IQR] mean (SD)	AIS female/male (N)	Definition controls	Control group (N)	Control age median [IQR] mean (SD)	Control female/male (N)	Outcome (AIS vs. control) median [IQR] mean (SD)
E-Selectin														
Shyu et al.	Prospective, observational	1997	n.a.	Taiwan	<24	ELISA	51	66 (12)	21/30	Subjects attending for routine health checkups. symptom-free/medication-free. Venous blood from 10 healthy and young laboratory staff	25	65 (13)	10/15	AIS: 47 ng/mL (SEM: 6) vs. HC: 39 ng/mL (SEM: 3), Young control: 41 ng/mL (SEM:3), *p* > 0.05
Simundic et al.	Prospective case–control	2004	n.a.	Croatia	<24	ELISA	67	72 (n.a.)	30/37	Controls were recruited from the CENTAR Health Center, Zagreb	76	52 (16)	52/24	AIS: 75 ng/mL (56) vs. controls: 62 ng/mL [range: 28–462], *p* = 0.002, OR: 1.022 (CI: 95% 1.008–1.037), *p* = 0.002
Licata et al.	Prospective, observational	2009	11/2002–01/2006	Italy	<12	ELISA	120	72 [64–82.5]	52/68	Patients admitted for any cause other than acute cardiovascular/cerebrovascular events	123	69 [65–83]	68/55	AIS: 2.25 ng/mL [2.0–4.0] vs. controls: 2 ng/mL [1.0–2.0], *p* < 0.001
Tuttolomondo et al.	Prospective, observational	2009	11/2006–10/2008	Italy	<12	ELISA	107	71 [63–80.5]	41/66	Matched for age (±3 years), sex, and cardiovascular risk factor prevalence	102	68 [63–80]	55/57	*All stroke:* 2.05 ng/mL [1.0–3.8] vs. controls: 2 ng/mL [1.0–2.0], *p* < 0.001, *LAAS*: 4.0 ng/mL [2.0–6.0], *LAC*: 3.0 ng/mL [2.0–5.0], *CEI*: 2.25 ng/mL [1.0–4.5], *ODE*: 3.5 ng/mL [1.8–3.6], *p* = 0.68
Abdulle et al.	Prospective, observational	2010	n.a.	United Arab Emirates	<24	ELISA	40	50.2 (9.5)	4/36	Hospitalized non-stroke patients	42	44.3 (14.9)	9/33	AIS: 15.1 ng/mL [1.7–126] vs. controls: 30.7 ng/mL [1.1–219], *p* = 0.136
P-Selectin														
Cha et al.	Prospective case–control	2003	06/2001–11/2001	South Korea	<24	FacScan	25	62.9 (10.5)	10/15	Patients with Parkinson’s disease, tension headache, or epilepsy.	24	61.7 (14.9)	12/12	Missing units, AIS: 120.1 (37.8) vs. controls: 75.3 (9.1), *p* < 0.01; Asymptomatic carotid stenosis: 118.9 (46.9) vs. control, *p* < 0.01
Licata et al.	Prospective, observational	2009	11/2002–01/2006	Italy	<12	ELISA	120	72 [64–82.5]	52/68	Patients admitted for any cause other than acute cardiovascular/cerebrovascular events	123	69 [65–83]	68/55	AIS: 4 ng/mL [2.0–6.3] vs. controls: 3.1 ng/mL [2.1–4.0], *p* = 0.004
Tuttolomondo et al.	Prospective, observational	2009	11/2006–10/2008	Italy	<12	ELISA	107	71 [63–80.5]	41/66	Matched for age (±3 years), sex, and cardiovascular risk factor prevalence	102	68 [63–80]	55/57	*All stroke:* 4.5 ng/mL [2.0–6.8] vs. controls: 3.1 ng/mL [2.1–4.0], *p* = 0.004; *LAAS*: 4.05 ng/mL [2.0–6.0], *LAC*: 4.0 ng/mL [2.2–7.0], *CEI*: 3.1 ng/mL [1.3–6.3], *ODE*: 1.55 ng/mL [1.0–5.9], *p* = 0.34
L-Selectin														
Fassbender et al.	Prospective case–control	1995	n.a.	Germany	4, 8, 10, 24	monoclonal two-sided immunoradiometric assay	22	72 (n.a.)	14/8	HC/Vascular risk factor (VRF)	22	69 (n.a.)	14/8	At 4 h: AIS: 933.33 ng/mL (SEM:72.48) vs. *HC*: 1063.18 ng/mL (SEM: 72.25), *VRF*: 865.49(SEM: 76.99), *p* > 0.05
Simundic et al.	Prospective case–control	2004	n.a.	Croatia	<24	ELISA	67	72 (n.a.)	30/37	Controls were recruited from the CENTAR Health Center, Zagreb	76	52 (16)	52/24	AIS: 963 ng/mL (395) vs. controls: 890 ng/mL [range: 490–2530], *p* = 0.043, OR: 1.00 (CI: 95% 0.999–1.001), *p* = 0.440

LAAS, Large Artery Atherosclerosis Stroke; LAC, Lacunar infarct; CEI, Cardio-Embolic Infarct; LVO, Large Vessel occlusive stroke; SVD, Small Vessel Disease; Afib, Atrial fibrillation; TIA, Transient Ischemic Attack; ODE, Other Determined Etiology; AIS, Acute Ischemic Stroke; h, hours; N, Number of patients; SD, Standard deviation; SEM, Standard Error of the Mean; IQR, Inter Quartile Range; n.a., not applicable; AUC, Area Under Curve; ROC, Receiver Operating Characteristic; OR, Odds Ratio; HC, Healthy Controls; ELISA, Enzyme-linked Immunosorbent Assay; Elecsys, ElectroChemi-Luminescence ImmunoAssay System; Eclia, ElectroChemiLuminescence ImmunoAssay; FacScan, Fluorescence Activated Cell Scanner; SIMOA, Single Molecule Array; HSP, Heat Shock Proteins; tPA, tissue Plasminogen Activator; E-Selectin, Endothelial-Selectin; P-Selectin, Platelet-Selectin; L-Selectin, Leukocyte-Selectin.

Higher P-Selectin values compared to controls were observed among 60% of studies, while 40% showed no difference (61, 62, 66). No differences between reported median E-Selectin levels were found for patients suffering LAAS, LAC, CEI, ODE (4.0 ng/mL, IQR: 2.0–6.0; 3.0 ng/mL, IQR: 2.0–5.0; CEI: 2.25 ng/mL, IQR: 1.0–4.5; ODE: 3.5 ng/mL, IQR: 1.8–3.6, respectively, Kruskal-Wallis test *p* = 0.68) (62) (Table 4).

#### 3.3.2 P-Selectin

Three studies measured P-Selectin and included 252 AIS patients and 249 controls who were admitted for reasons other than cerebrovascular events, i.e., Parkinson’s disease, tension headache, and epilepsy, age, sex, and presence of cardiovascular risk factors matched patients (61, 62, 71). Studies were published between 2003 and 2009 and mean blood draw time ranged between from within <12 h to within <24 h after symptom onset. Measurement of P-Selectin was accomplished using ELISA in two studies carried out in Italy, whereas a South Korean study used Fluorescence-activated cell sorting (FacScan) (Table 4). Two of the studies were of good quality and one study revealed fair quality (Supplementary Table S3).

All studies reported higher P-Selectin values in stroke patients compared to controls (61, 62, 71). However, no differences between median P-Selectin levels were found for different stroke etiologies (LAAS: 4.05 ng/mL, IQR: 2.0–6.0; LAC: 4.0 ng/mL, IQR: 2.2–7.0; CEI: 3.1 ng/mL, IQR: 1.3–6.3; ODE: 1.55 ng/mL, IQR: 1.0–5.9, Kruskal-Wallis *p* = 0.34) (61, 62). P-Selectin was also reported as elevated in AIS patients, as well as in asymptomatic atherosclerotic stenosis patients compared to healthy controls (*p* < 0.01) (71). P-Selectin levels did not differ between AIS patients and asymptomatic carotid stenosis patients in the same study (71).

#### 3.3.3 L-Selectin

Through our literature research, two articles were found on L-Selectin (65, 66). (Table 3) In total, 89 AIS patients and 98 healthy control patients were recruited from the CENTAR, Health Center Zagreb, Croatia (Table 4). Both studies showed fair quality according to the NOS (Supplementary Table S3).

One study measured blood L-Selectin levels in patients experiencing stroke and HC using ELISA samples drawn within 24 h of AIS symptom onset. Higher concentrations of L-selectin in AIS patients compared to the controls were observed (963 ng/mL, SD: 395 vs. 890 ng/mL, range: 490–2530, *p* = 0.043) (66). However, the other study reported contrary results using a monoclonal two-sided immunoradiometric assay. No differences in L-Selectin levels between AIS patients and healthy controls at 4 h after symptom onset were observed (933.33 ng/mL, standard error of the mean (SEM): 72.48 vs. 1063.18 ng/mL, SEM: 72.25, *p* > 0.05). Furthermore, slight changes in L-Selectin levels were observed 8 and 10 h after the onset of symptoms while no differences between various time points and HC were observed (65). One study showed that L-Selectin levels decreased with a higher number of risk factors (smoking, hypertension, diabetes mellitus, or hypercholesterolemia) one risk factor: 777.35 ng/mL (SEM:114.03) vs. four risk factors: 692.75 ng/mL (SEM: 112.60) (65).

### 3.4 Glial and neuronal markers

#### 3.4.1 NSE

Ten studies evaluating NSE comprising 931 AIS patients and 527 control subjects (HC/matched controls: *n* = 329, stroke mimics: *n* = 198) were included in this review. The controls encompassed five studies with HC, three studies with control patients with conditions other than neurological diseases, and two studies with stroke mimics as the controls (53, 55, 72–79) (Table 4). Most of the studies were carried out in Germany (*n* = 2) and South Korea (*n* = 2), followed by Turkey (*n* = 1), USA (*n* = 1), Ireland (*n* = 1), Netherlands (*n* = 1), Spain (*n* = 1), and China (*n* = 1). Publication years ranged between 1997 and 2018. Reported blood drawing time ranged from 4 h to <24 h. In 60% of studies, NSE was measured using ELISA. Other techniques applied were radioimmunoassay as well as antibody-based arrays and monoclonal two-sided immunoradiometric assay (Table 5). Study quality, evaluated with the NOS was found to be of good quality in 20%, and fair quality in 80% of studies (Supplementary Table S4).

**Table 5:** Glial and neuronal biomarkers.

Author	Study type	Study year	Study period	Origin of the study	Blood drawing median [IQR] mean (SD) in h	Measurement method	AIS (N)	AIS age, median [IQR] mean (SD)	AIS female/male (N)	Definition controls	Control group (N)	Control age median [IQR] mean (SD)	Control female/male (N)	Outcome (AIS vs. control) median [IQR] mean (SD)
NSE—controls other than stroke mimics														
Cunningham et al.	Prospective case–control	1991	n.a.	Ireland	24, 48, 72, 96	Radioimmunoassay	24	69.58 (12.39)	14/10	Patients who had cardiac or respiratory disease (free from neurological disorders)	57	51.06 (16.7)	27/30	AIS: 25.38 μg/L (18.6) vs. controls: 8.34 μg/L (2.65), *p* = n.a.
Fassbender et al.	Prospective case–control	1997	n.a.	Germany	4, 8, 10, 24, 72	Monoclonal two-sided immunoradiometric assay	24	72 (n.a.)	14/10	Age- and sex-matched HC	24	66 (n.a.)	13/11	AIS: NSE-levels at 4 h [14.63 ng/mL (SEM:2.30)], 8 h [17.31 ng/mL (SEM:2.69)], 10 h [18.86 ng/mL (SEM:4.70)], 24 h [14.98 ng/mL (SEM:1.95)] vs. controls 9.11 ng/mL (SEM:0.73), *p* ≤ 0.05
Missler al.	Prospective case–control	1997	02/95–10/95	Germany	<24	ELISA	41	65 (n.a.)	12/32	HC	98	39.3 (11.7)	48/50	AIS: 22.7 μg/L (10.1) vs. HC:11.1 μg/L (4.7), *p* ≤ 0.05
Sulter et al.	Prospective case–control	1998	n.a.	Netherlands	<24	Radioimmunoassay	41	71.97 (9.58)	22/19	HC	27	n.a.	n.a.	Total anterior circulation syndrome 10.3 ng/mL (SEM:1.0); partial anterior syndrome 7.8 ng/mL (SEM: 0.6), Lacunar syndrome 7.3 ng/mL (SEM: 0.56), Control: 7.1 ng/mL (SEM: 0.3); TACS vs. PACS, *p* = 0.046, TACS vs. LAC, *p* = 0.061
Oh et al.	Prospective case–control	2003	04/2000–12/2001	South Korea	12.8 (8.4)	ELISA	81	66.7 (13.8)	39/42	Age-and sex-matched HC	81	63.8 (11.1)	37/40	AIS: 13.0 ng/dL (5.4) vs. HC: 6.3 ng/dL (1.6), *p* ≤ 0.05
Wu et al.	Prospective case–control	2004	10/98–10/2000	China	<10.1 [4.2–16.8]	ELISA	38	66.2 (12.5)	17/21	Randomly chosen HC	27	40.3 (17.1)	10/17	AIS: 18.48 ng/mL (16.61) vs. HC: 9.00 ng/mL (2.70), *p* < 0.001
Gojska-Grymajlo et al.	Prospective case–control	2018	n.a.	Poland	<24	ELISA	67	69.3 (12.3)	31/36	Co-morbidity-matched and with no previous history of stroke	15	62.2 (3.7)	6/9	AIS: 25.01 ng/mL (19.64) vs. controls: 12.39 ng/mL (7.98), *p* = 0.0018
NSE—stroke mimics														
An et al.	Prospective, observational	2013	09/2010–10/2010	South Korea	11 [7–16]	ELISA	188	66 (11)	87/101	Stroke mimics	90	61 (9)	53/37	AIS: 6.2 ng/mL [5.3–7.5] vs. stroke mimics: 4.2 ng/mL [2.1–6.9], *p* = 0.096
Cakmak et al.	Prospective case–control	2014	n.a.	Turkey	9.2 (9)	ELISA	38	66.1 (12.8)	18/20	Stroke mimics	30	64.6 (12.4)	10/20	AIS: 32.671 mg/L (30.42) vs. stroke mimics: 17.417 mg/L (7.08), *p* = 0.005 Cut-off value: 18 μg/L, 61% sensitivity, 53% specificity, 52% NPV, and 62% PPV.
Bustamante et al.	Prospective, observational, multicenter	2017	08/2012–11/2013	Spain	<6	Antibody-based array (Search Light Technology Aushon BioSystems, Billerica)	389	n.a.	n.a.	Stroke mimics	78	n.a.	n.a.	AIS: 4.07 ng/mL [3.72–4.47] vs. stroke mimics: 4.04 ng/mL [3.74;4.28], *p* = n.a.
GFAP—controls other than stroke mimics														
Ren et al.	Prospective case–control	2016	n.a.	China	10 [4–24]	ELISA	79	61.1 (13.33)	30/49	HC	57	58.93 (9.82)	37/30	AIS: 0.02 ng/mL [0.004–0.08] vs. HC: 0.004 ng/mL [0.004–0.02], *p* < 0.003 *ROC-analysis*: 0.71 (95% CI: 0.63–0.79)
Ekingen et al.	Prospective case–control	2017	12/2011–11/2012	Turkey	3 [1.5–3.2.]	ELISA	40	70.72 (9.8)	21/19	HC	50	n.a.	n.a.	AIS normal cCT: 39.80 ng/mL [26.94–44.53] vs. HC: 24.69 ng/mL [10.66–36.66], *p* = n.a. AIS normal cCT vs. AIS with abnormal cCT: 39.61 ng/mL [28.63–43.56], *p* = 0.924 AIS (all) vs. HC, *p* < 0.001 *ROC-analysis*: 70.59% sensitivity and 70% specificity, AUC = 0.684 (95% CI: 0.558–0.792), *p* = 0.0213, cutoff value = 33.24 ng/mL
Katsanos et al.	Prospective case–control	2017	12/2013–12/2015	Greece	<6	ELISA	121	77.3 (9.1)	59/62	HC	79	39.5 (11.8)	16/63	AIS: 0.11 ng/mL [0–0.27] vs. HC: 0 ng/mL [0–0.22], *p* = n.a.
Luger et al.	Prospective, observational study, multicenter	2017	04/2012–09/2013	Germany	AIS: 1.9 (0.27)	ECLIA	146	74.5 (11.9)	73/73	HC	115	n.a.	n.a.	AIS: 0.01 μg/L [0.01–0.01] vs. HC: Values <0.03 μg/L, *p* = n.a.
Ferrari et al.	Longitudinal, prospective, observational study	2023	08/2019–02/2021	Italy	<24	SIMOA	34	n.a.	n.a.	HC	20	48.15 (9.57)	11/9	AIS: 601.3 [2432.6] vs. HC: 87.9 pg/mL [72.9], *p* < 0.0001
Kowalski et al.	Prospective case–control	2023	08/2021–04/2022	USA	2.22 (n.a.)	SIMOA	4	65.7 (20.02)	n.a.	HC	21	21 [18–26]	11/10	AIS: 195.22 pg./mL [range: 52.77–1526.74] vs. HC: 80.37 pg/mL [range: 56.43–132.86], *p* = n.a.
GFAP—stroke mimics														
Foerch et al.	Prospective, observational study,	2012	06/2009–05/2010	Germany	<4.5	ECLIA	163	75.3 (13.4)	84/79	Stroke mimics	3	44.3 (23.5)	1/2	AIS: 0.08 μg/L [0.02–0.14] vs. stroke mimics: 0.19 μg/L [0.16–0.21], *p* = n.a.
An et al.	Prospective, observational	2013	09/2010–10/2010	South Korea	11 [7–16]	ELISA	188	66 (11)	87/101	Stroke mimics	90	61(9)	53/37	AIS: 52 (38.2) vs. stroke mimics: 14 (15.6), *p* < 0.029; units missing
Katsanos et al.	Prospective case–control	2017	12/2013–12/2015	Greece	<6	ELISA	121	77.3 (9.1)	59/62	Stroke mimics	31	58.1 (11)	n.a.	AIS: 0.11 ng/mL [0–0.27] vs. stroke mimics: 0.07 ng/mL [0–0.24], *p* = n.a.
Luger et al.	Prospective, observational study, multicenter	2017	04/2012–09/2013	Germany	AIS: 1.9 (0.27)	ECLIA	146	74.5 (11.9)	73/73	Stroke mimics	11	73.5 (10)	5/6	AIS: 0.01 μg/L [0.01–0.01] vs. stroke mimic: 0.01 μg/L [0.01–0.01], *p* = n.a.
					Stroke mimics: 1.37 (0.83)									
Luger et al.	Prospective, observational study, two-center investigation	2020	10/2014–02/2016	Norway Østfold county	<6	ELISA	23	81.8 (17.2)	17/6	Stroke mimics	14	60.4 (17.2)	10/4	AIS: 30.0 ng/L [12.0–62.0] vs. stroke mimics: 12.0 ng/L [7.5–27.0], *p* = n.a.
				Frankfurt			148	73.0 (15.5)	85/63		2	86.5 (5.0)	2/0	AIS: 28.5 ng/L [16.0–58.8] vs. stroke mimics: 111 ng/L (n.a.), p = n.a.
Kalra et al.	Prospective case–control	2021	11/2018–05/2019	India	<12	SIMOA	75	64 (14)	28/47	Stroke mimics	10	61 (12)	5/5	AIS: 0.18 μg/L [0.11–0.38] vs. stroke mimics: 0.14 μ/l [0.09–0.26], *p* = n.a.
Jaeger et al.	Prospective case–control	2023	05/2017–03/2020	Norway	4 [3–7]	SIMOA	131	73(13)	56/75	Stroke mimics	114	65 (17)	56/58	AIS: 268 pg/mL [171–514] vs. stroke mimics: 192 pg/mL [109–386], *p* = n.a. *AIS vs. stroke mimic, ROC-analysis*: GFAP: 0.47 (95%CI: 0.40–0.55)
S100														
Büttner et al.	Prospective case–control	1997	n.a.	Germany	<24	Two-site radioimmunoassay	26	71.7 (14.2)	15/11	HC	26	86.3 (8.6)	15/11	AIS: 0.25 μg/L (SEM:0.15) vs. controls: S100 < 0.2 μg/L, *p* ≤ 0.05
Fassbender et al.	Prospective case–control	1997	n.a.	Germany	4, 8, 10, 24, 72	Monoclonal two-sided immune-radiometric assay	24	72 (n.a.)	14/10	Age- and sex-matched HC	24	66 (n.a.)	13/11	AIS S100-levels at 8, 10, 24 h vs. control, *p* ≤ 0.05; “S-100 protein was detectable in none of the control subjects”
Missler et al.	Prospective case–control	1997	02/1995–10/1995	Germany	<24	ELISA	44	65 (n.a.)	12/32	HC	98	39.3 (11.7)	48/50	AIS: 0.21 μg/L (0.64) vs. HC: 0.07 μg/L (0.58), *p* ≤ 0.05
Rainer et al.	Prospective case–control	2007	n.a.	China	10 [9–14]	ELISA	118	n.a.	n.a.	Age- and sex-matched HC	20	n.a.	n.a.	AIS: 0.14 μg/L [0.05–0.47] vs. HC: 0.12 μg/L [0.0–0.104], *p* = n.a. OR: 12.42 95% (CI: 1.50–103.02; *p* < 0.001).
Üstündağ et al.	Prospective case–control	2011	03/2008–09/2009	Turkey	7.3 (5.2)	ELISA	90	65.1 (10)	55/35	Age and sex-matched controls, (Admission due to headache, osteoarthritis, or minor trauma)	16	63.5 (3.1)	9/7	*Atherothrombotic stroke, 0.47 ng/mL (0.45); *Cardioembolic stroke, 0.47 ng/mL (0.31); Lacunar stroke, 0.10 ng/mL (0.07); **TIA, 0.18 ng/mL (0.22) vs. Control: 0.05 μg/L (0.02), *p < 0.001; **p > 0.05 *All stroke*: OR 15.24; 95% CI: 4.35, 53.38; *p* < 0.001
S100B—controls other than stroke mimics														
Reynolds et al.	Prospective case–control	2003	11/1999–02/2003	USA	<12	ELISA	38	n.a.	n.a.	HC	214	n.a.	n.a.	AIS: 44.6 ng/L [5.46–96.56] vs. HC: 19.98 ng/L [0.0–67.92], *p* = n.a. OR: 1.28 (95% CI: 0.47–2.33)
Kim et al.	Prospective case–control	2010	04/2007–08/2007	South Korea	<6	Triage^®^ Meter POCT instrument (Biosite, Inc.).	89	66.6 (11.8)	39/50	HC	57	43.8 (12)	32/25	AIS: 103.1 pg/mL (13.6) vs. HC: 188.6 pg/mL (14.1), *p* < 0.001
S100B—stroke mimics														
Laskowitz et al.	Prospective case–control	2009	07/2002–06/2005	USA	9.3 [4.5–18.2]	Point-of-care fluorescence immunoassay	345	n.a.	n.a.	Stroke mimics	425	n.a.	n.a.	AIS: 610 pg/mL vs. stroke mimics: 316 pg/mL, *p* < 0.005
Montaner et al.	Prospective, observational	2010	n.a.	Spain	<24	ELISA	915	72.63 (12.46)	443/471	Stroke mimics	90	69.57 (17.13)	34/65	AIS: 62.76 pg/mL [32.59;130.47] vs. stroke mimics: 65.04 pg/mL [35.78;137.98], *p* = 0.878
Glickman et al.	Prospective, observational	2011	n.a.	USA	3.5 (8)	ELISA	34	65.2 (16.2)	22/12	Stroke mimics	29	50.9 (19.1)	20/9	Missing units: AIS: 124.7 (123.1) vs. stroke mimics: 58 (41.7), *p* = 0.003; OR: 3.62, 95% (CI: 1.29–10.15)
Vanni et al.	Prospective, observational	2011	06/2006–09/2006	Italy	6 (7)	Triage^®^ Meter point-of-care instrument	87	74 (11)	34/53	Stroke mimics	68	69 (16)	31/38	AIS: 251 pg/mL (0) vs. stroke mimics: 251 pg/mL (4), *p* = 0.495
An et al.	Prospective, observational	2013	09/2010–10/2010	South Korea	11 [7–16]	ELISA	188	66 (11)	87/101	Stroke mimics	90	61 (9)	53/37	AIS: 30.4 pg/mL [0–115.2] vs. stroke mimics: 2.3 pg/mL [0.0;20.6], *p* < 0.0001
Park et al.	Prospective, observational	2013	09/2008–12/2010	South Korea	11.4 (9.7)	ELISA	111	n.a.	n.a.	Stroke mimics	127	63 (9)	77/50	*Blood draw <6 h:* AIS: 111 pg/mL (210) vs. stroke mimics: 21 pg/mL (55), *p* < 0.001 *Blood draw 6–24 h*: AIS: 124.2 pg/mL (223.3) vs. stroke mimics: 21.0 pg/mL (55.7), *p* < 0.001 OR: 1.01 (95% CI: 1.00–1.01), *p* < 0.003 *ROC-analysis*: AUC: 0.70 (sensitivity: 0.54, specificity: 0.835), *p* < 0.001
Cakmak et al.	Prospective case–control	2014	n.a.	Turkey	9.2 (9)	ELISA	38	66.1(12.8)	18/20	Stroke mimics	30	64.6 (12.4)	10/20	AIS: 197.50 pg/mL (242.11) vs. stroke mimics: 62.27 pg/mL (11.87), *p* = 0.001
Bustamante et al.	Prospective, observational, multicenter	2017	08/2012–11/2013	Spain	<6	Antibody-based array (Search Light Technology, Aushon BioSystems, Billerica)	389	n.a.	n.a.	Stroke mimics	78	n.a.	n.a.	AIS: 4.06 pg/mL (2.54–4.7) vs. stroke mimics: 3.85 pg/mL [2.62–5.18], *p* = n.a.

LAAS, Large Artery Atherosclerosis Stroke; LAC, Lacunar infarct; CEI, Cardio-Embolic Infarct; LVO, Large vessel occlusive stroke; SVD, Small Vessel Disease; Afib, Atrial fibrillation; TIA, Transient Ischemic Attack; ODE, Other Determined Etiology; AIS, Acute Ischemic Stroke; h, hours; N, Number of patients; SD, Standard deviation; SEM, Standard Error of the Mean; IQR, Inter Quartile Range; n.a., not applicable; AUC, Area Under Curve; ROC, Receiver Operating Characteristic; OR, Odds Ratio; HC, Healthy Controls; ELISA, Enzyme-linked Immunosorbent Assay; Elecsys, ElectroChemiLuminescence ImmunoAssay System; Eclia, ElectroChemiLuminescence ImmunoAssay; FacScan, Fluorescence Activated Cell Scanner; SIMOA, Single Molecule Array; HSP, Heat Shock Proteins; tPA, tissue Plasminogen Activator; NSE, Neuron Specific Enolase; GFAP, Glial Fibrillary Protein; cCT, cranial Computer Tomography; TACS, Total Anterior Circulation Syndrom; PACS, Partial Anterior Circulation Syndrom; PPV, positive predictive value; NPV, negative predictive value.

##### 3.4.1.1 AIS vs. healthy controls/patients with other than neurological diseases

Higher NSE concentrations in AIS patients compared to controls were reported in 57% of the studies (74, 76, 77, 79). In two studies, no significant differences were found between AIS patients and controls (72, 75). One study described that NSE levels in AIS patients were elevated at 8, 10, and 24 h after symptom onset (*p* ≤ 0.05), but not 4 h after symptom onset (73).

##### 3.4.1.2 AIS vs. controls and stroke mimics

Two studies revealed no differences in NSE levels between AIS patients and controls (53, 55). One study showed higher NSE levels in AIS patients compared to stroke mimics (32.671 mg/L, SD: 30.42 vs. 17.417 mg/L, SD: 7.08, *p* = 0.005) (78). An ROC analysis revealed an area under curve (AUC) of 0.67 for NSE (bootstrap 95% CI: 0.55–0.80). The best cut-off value for NSE was found to be 18 μ/L, with a sensitivity of 61%, specificity of 53%, negative predictive value (NPV) of 52%, and positive predictive value (PPV) of 62% (78).

##### 3.4.1.3 Etiology-, outcome-, severity related NSE levels in AIS patients

In AIS patients with cortical and non-cortical infarcts, contrasting results in NSE values were found by infarct location (73). One study reported a difference in NSE levels, whereas another study showed no difference (73, 77). However, patients with total anterior cerebral infarction (TACI) had higher serum NSE levels on admission compared to patients with partial anterior cerebral infarction (PACI) but not LAC (10.3 ng/mL, SEM: 1.0; 7.6 ng/mL, SEM: 0.5; 7.8 ng/mL, SEM: 0.6, respectively) (75). Another study reported higher NSE levels in 71% of patients with LAC as well as 53% of TACI patients compared to controls (76). Additionally, patients with cortical infarctions presented with higher NSE levels when hyperglycemia was persistent (11.2 ng/mL, SEM: 0.8 vs. 7.2 ng/mL, SEM: 0.5, *p* = 0.0008). This was not seen in patients experiencing lacunar stroke (75). Three studies showed a positive correlation between NSE levels and lesion volume on brain imaging (*p* ≤ 0.05) (72, 74, 76). When comparing infarct volume of less than 5 cm (3) with >5 cm (3), no difference in NSE concentrations was observed (73). Patients with chronic atrial fibrillation showed higher day one NSE concentrations than patients without (37.39 ng/mL, SD: 9.37 vs. 24.49 ng/mL, SD: 23.16, *p* = 0.0018) (79).

#### 3.4.2 GFAP

For the analysis of GFAP, 11 studies were eligible for inclusion comprising 1,152 AIS patients and 617 control patients (HC: *n* = 342, stroke mimics: *n* = 275) (15, 16, 57, 80–85) (Table 4). Most of the studies were carried out in Germany (*n* = 3) followed by China (*n* = 1), Greece (*n* = 1), Norway (*n* = 1), India (*n* = 1), USA (*n* = 1), Italy (*n* = 1), South Korea (*n* = 1), and Turkey (*n* = 1) and publication years ranged between the 2013 and 2023. Almost half of the studies (45.5%) of studies used ELISA, 36.4% used SIMOA, and two used electro-chemiluminometric immune assay. Blood drawing time ranged between 1.9 h and < 24 h after symptom onset (Table 5). Risk of bias assessment revealed four studies with good quality while seven studies were found to be of fair quality. Poor quality was not observed (Supplementary Table S4).

##### 3.4.2.1 AIS vs. healthy controls

Six studies compared AIS patients with patients who experienced stroke albeit two studies investigated both healthy controls and stroke mimics. Half of the studies showed higher GFAP levels in AIS patients compared to controls (*p* ≤ 0.05) (81, 85, 86). One study showed higher levels of GFAP in AIS patients compared to HC but statistical testing for differences was not performed (195.22 pg./mL, range: 52.77–1526.74 vs. 80.37 pg/mL, range: 56.43–132.86) (57). Two studies showed comparable GFAP concentrations between both groups (82, 83). GFAP levels were higher in brain CT image-confirmed AIS patients as well as patients with clinically suspected stroke and normal CT, than in HC. In addition, it was reported that no difference was found in patients with suspected stroke without brain CT imaging signs of stroke compared to patients with positive CT findings (86). When applying a cut-off value of 33.24 ng/mL for GFAP, ROC analysis showed a sensitivity of 70.59% and a specificity of 70% (AUC = 0.684, 95% CI: 0.558–0.792) for the differentiation between AIS and HC. Furthermore, no association between stroke severity and GFAP levels was reported (*r* = 0.164, *p* = 0.311) (86).

##### 3.4.2.2 AIS vs. stroke mimics

Seven studies compared AIS patients with stroke mimics (15, 16, 53, 80, 82–84). Interestingly, lower GFAP concentrations in AIS versus stroke mimic patients on admission were observed in one study (0.08 μg/L, IQR: 0.02–0.14 vs. 0.19 μg/L, IQR: 0.16–0.21) (80). Contrary results were obtained in another study (*p* = 0.029) (53). Higher GFAP concentrations were observed in two studies, however, no statistical comparisons of the two groups were reported (16, 84). Three studies reported comparable GFAP levels in AIS patients and stroke mimic patients (15, 82, 83). GFAP was not found to differentiate AIS from stroke mimics per a reported AUC of 0.47 (95% CI: 0.40–0.55) (16).

#### 3.4.3 S100

Five studies measuring S100 were eligible for this review (73, 74, 87–89). In total, 302 AIS patients and 184 HC/matched controls were included and reported blood drawing time between 4 h and < 24 h after symptom onset. Sixty percent of the studies were conducted in Germany (*n* = 3), followed by China (*n* = 1) and Turkey (*n* = 1). S100 was measured using ELISA (*n* = 3) or a two-site radioimmunoassay (*n* = 2). The studies were published between 1997 and 2011 (Table 5). Two studies revealed good quality and three studies fair quality when evaluated with NOS (Supplementary Table S4).

In 60% of the studies, higher serum S100 protein levels were observed in AIS patients compared to HC and/or an age- and sex-matched control group (73, 74, 87). Patients with CEI and atherothrombotic stroke had higher S100 values than age and sex-matched controls (0.47 ng/mL, SD: 0.31; 0.47 ng/mL, SD: 0.45 vs. 0.05 μg/L, SD: 0.02, respectively, *p* < 0.001) (89). A positive correlation between S100 concentrations and infarct size was detected (*r* = 0.75, *p* < 0.001) (74). A higher S100 concentration in MCA infarction patients compared to HC was found in another study (87). Initially higher concentrations of S100 were more associated with a worse functional outcome albeit a significant correlation was not reported (87).

S100 protein emerged as a predictor of post-stroke mortality as an increase of 0.735 μg/L led to an increased post-stroke mortality of 110% (88). However, one study showed increasing S100 in stroke patients depending on the time of blood collection (12.5% at hour 4; 20.8% at hour 8, 37.5% at hour 10, 60.9% at hour 24, and 57.1% at hour 72) (73).

#### 3.4.4 S100B

Our literature search revealed 10 studies on S100B (34, 42, 46, 50, 51, 53, 55, 78, 90, 91). In total, 2,234 patients experiencing stroke and 1,208 controls (HC: *n* = 271, stroke mimics: *n* = 937) were included. Control groups included stroke mimics (80% of studies) and two studies enrolled HC. To measure S100B, ELISA was used in six studies, a POCT in three studies, and an antibody array in one study. Blood samples were taken between 3.5 h and < 24 h after the onset of stroke symptoms. Most of the studies were carried out in South Korea (*n* = 3) followed by Spain (*n* = 2), USA (*n* = 3), Italy (*n* = 1), and Turkey (*n* = 1) (Table 5). In the risk of bias assessment, three of the included studies showed good quality and seven showed fair quality (Supplementary Table S4).

##### 3.4.4.1 AIS vs. healthy controls

The literature search revealed two studies with contrary results as one study reported lower S100B levels in AIS patients compared to controls (103.1 pg/mL, SD: 13.6 vs. 188.6 pg/mL, SD: 14.1, *p* < 0.001) while another study found higher mean S100B levels in AIS patients compared to controls (44.6 ng/L, IQR: 5.46–96.56 vs. 19.98 ng/L, IQR: 0.0–67.92) (34, 46).

##### 3.4.4.2 AIS vs. stroke mimics

Five studies showed higher S100B levels in AIS patients compared to stroke mimics (51, 53, 78, 90, 91). However, two studies did not reveal any differences in S100B levels between the two groups while one study reported similar GFAP levels between the groups (42, 50, 55). One study showed a weak to moderate predictive value of S100B for the discrimination of AIS (AUC: 0.70, cut-off value: 23.5 pg./mL, sensitivity: 54.0%, specificity: 83.5%, *p* < 0.001) (90). However, excellent discrimination was found by another study using a cut-off value of 65 μg/L resulting in an AUC of 0.89 (bootstrap 95% CI: 0.81–0.96), a sensitivity of 87%, a specificity of 72%, and an NPV as well as PPV of 81% (78). Stroke severity measured with the NIHSS was positively correlated with S100B levels (*r* = 0.45, *p* ≤ 0.05) in the study with moderate predictive value (90).

### 3.5 Biomarkers of cardiac dysfunction

#### 3.5.1 BNP

BNP was measured in seven studies, two of which were performed in Turkey, and one each in South Korea, Italy, USA, Japan, and Spain (34, 42, 50, 51, 92–94). There were a total of 1,313 AIS patients and 373 control patients (HC: *n* = 2286, stroke mimics: *n* = 187). Of those, HC were examined in three studies, three studies included stroke mimics and one study included control patients with hypertension. Blood drawing time ranged between 3.5 and < 24 h after the onset of symptoms. Publication years ranged between 2004 and 2011. The most used method for measuring BNP was a POCT followed by ELISA, microparticle immunoassay, and a highly sensitive two-site immunoradiometric assay (Table 6). The quality of studies was found to be good in one study and six studies showed fair quality according to the NOS (Supplementary Table S5).

**Table 6:** Biomarkers of cardiac dysfunction.

Author	Study type	Study Year	Study period	Origin of the study	Blood drawing median [IQR] mean (SD) in h	Measurement method	AIS (N)	AIS age, median [IQR] mean (SD)	AIS female/male (N)	Definition controls	Control group (N)	Control age median [IQR] mean (SD)	Control female/male (N)	Outcome (AIS vs. control) median [IQR] mean (SD)
BNP—controls other than stroke mimics														
Nakagawa et al.	Prospective case–control	2004	01/2000–12/2001	Japan	<24	Highly sensitive 2-site immunoradiometric assay (SHIONORIA BNP, Shionogi & Co., Ltd.)	88	64.22 (10.71)	29/59	HC (medical staff)	79	62.54 (7.67)	33/46	*AIS ± Afib:* 186.3 pg/mL (153.6); *AIS without Afib*: 59.5 pg/mL (49.3) vs. *Afib without stroke*: 68.1 pg/mL (53.5), HC: 8.1 pg/mL (4.5) Afib without stroke vs. HC, *p* < 0.0021; Essential HT: 22.2 pg/mL (17.2) vs. HC, *p* = 0.382; AIS without Afib vs. HC, *p* = 0.0012; AIS + Afib vs. HC, *p* ≤ 0.05
Cakir et al.	Prospective, observational	2010	11/2006–11/2007	Turkey	<12	Triage BNP Test (Biosite Inc., San Diego, California, United States).	60	62.6 (14.71)	36/24	Hypertension (HT) patients without stroke	20	55 (11.2)	11/9	*Stroke with HT*: 168.8 pg/mL (223.9); *Stroke without HT*: 85.0 pg/mL (75.1) vs. 84.8 pg/mL (178.3), *p* ≤ 0.05 (for all group comparisons)
Kim et al.	Prospective case–control	2010	04/2007–08/2007	South Korea	6	Triage^®^ Meter POCT instrument (Biosite, Inc.).	89	66.6 (11.8)	39/50	HC	57	43.8 (12)	32/25	AIS: 90.8 pg/mL (156.4) vs. HC: 11.3 pg/mL (6.1), *p* < 0.001
Saadet Sayan, Dilcan Kotan	Prospective case–control	2016	05/2013–05/2015	Turkey	<24	Microparticle immunoassay (i1000 Architect, Abbott Laboratories)	40	66.03 (9.95)	22/18	HC	30	51.54 (7.67)	22/8	AIS: 284.16 pg/mL (382.79) vs. HC: 25.29 pg/mL (13.47), *p* < 0.001; *LAA*: 142.90 pg/mL (10); *CEI*: 375 pg/mL (53); *SVD*: 163.87 pg/mL (22); *ODE*: 317.32 pg/mL (33), *p* > 0.05
BNP—stroke mimics														
Montaner et al.	Prospective, observational	2010	n.a.	Spain	<24	ELISA	915	72.63 (12.46)	443/471	Stroke mimics	90	69.57 (17.13)	34/65	AIS: 66.6 pg/mL [21.61–160.11] vs. stroke mimics: 49.1 pg/mL [0.0–132.4], *p* = 0.101
Glickman et al.	Prospective, observational	2011	n.a.	USA	3.5 (8)	ELISA	34	65.2 (16.2)	22/12	Stroke mimics	29	50.9 (19.1)	20/9	Missing units: AIS: 244.3 (395.6) vs. stroke mimics: 77.0 (194.8), *p* = 0.019; OR: 2.17 (0.90–5.24), *p* = 0.084
Vanni et al.	Prospective, observational	2011	06/2006–09/2006	Italy	6 (7)	Triage^®^ Meter point-of-care instrument	87	74 (11)	34/53	Stroke mimics	68	69 (16)	31/38	AIS: 175 pg/mL (368) vs. stroke mimics: 43 pg/mL (50), *p* = 0.001
NT-pro-BNP—controls other than stroke mimics														
Giannakoulas et al.	Prospective, observational	2005	n.a.	Greece	<24	EIA (Enzyme Immunoassay)	30	73.8 (1.1)	14/16	Age-sex-matched HC	30	71.5 (1.4)	14/16	AIS: 129.9 fmol/mL (SEM:9.9) vs. HC: 90.8 fmol/mL (SEM:6.3), *p* ≤ 0.05
Iltumur et al.	Prospective case–control	2006	10/2003–05/2004	Turkey	<24	Elecsys	57	64.5 (11.3)	37/20	Age- and gender-matched HC	57	61.3 (6.09)	36/21	AIS: 3,935 pg/mL (6274) vs. HC: 42 pg/mL (29), *p* < 0.001
NT-proBN—stroke mimics														
Bustamante et al.	Prospective, observational, multicenter	2017	08/2012–11/2013	Spain	<6	Antibody-based array (Search Light Technology, Aushon BioSystems, Billerica)	941	n.a.	n.a.	Stroke mimics	193	n.a.	n.a.	AIS: 5.95 pg/mL (SEM: 1.45) vs. stroke mimics: 5.15 pg/mL (SEM: 1.46), *p* = n.a. *ROC analysis*: sensitivity: 76.9%, specificity: 43.5% AUC = 0.742 (95% CI: 0.686–0.797), *p* < 0.0001

LAAS, Large Artery Atherosclerosis Stroke; LAC, Lacunar infarct; CEI, Cardio-Embolic Infarct; LVO, Large vessel occlusive stroke; SVD, Small Vessel Disease; Afib, Atrial fibrillation; TIA, Transient Ischemic Attack; ODE, Other Determined Etiology; AIS, Acute Ischemic Stroke; h, hours; N, Number of patients; SD, Standard deviation; SEM, Standard Error of the Mean; IQR, Inter Quartile Range; n.a., not applicable; AUC, Area Under Curve; ROC, Receiver Operating Characteristic; OR, Odds Ratio; HC, Healthy Controls; ELISA, Enzyme-linked Immunosorbent Assay; Elecsys, ElectroChemiLuminescence ImmunoAssay System; Eclia, ElectroChemiLuminescence ImmunoAssay; FacScan, Fluorescence Activated Cell Scanner; SIMOA, Single Molecule Array; HSP, Heat Shock Proteins; tPA, tissue Plasminogen Activator; BNP, Brain Natriuretic Peptide; NT-proBNP, N-terminal pro-B-type natriuretic peptide.

##### 3.5.1.1 AIS vs. healthy controls/patients with other than neurological diseases; etiology-, outcome-, severity related NSE levels in AIS patients

Higher BNP levels in AIS patients compared to controls were found in all of the studies (34, 92–94). BNP levels were not correlated with infarct size or stroke severity measured by the NIHSS but positively correlated with mean arterial pressure (MAP) (94). Also, in one study, a positive correlation between MAP and BNP levels but no correlation between stroke severity and BNP levels or infarct size were detected (94). Positive correlations between mean BNP levels and age as well as MAP were also found in one study, whereas no correlation between BNP levels and ventricular ejection fraction was detected (93).

Furthermore, BNP levels in AIS patients with atrial fibrillation (AF) as well as AIS patients without AF were compared to healthy controls. The highest BNP concentrations were found in AIS + AF patients while AIS patients without AF and AF patients without AIS had similar BNP levels (AIS + AF: 186.3 pg/mL, SD: 153.6; AIS without AF: 59.5 pg/mL, SD: 49.3 vs. AF without AIS: 68.1 pg/mL, SD: 53.5). However when comparing these subgroups with HC, BNP levels were lower in HC (92). In addition, BNP levels and MAP on admission were positively correlated in AIS patients without AF (*r* = 0.34). In contrast, an inverse correlation of BNP and MAP in AIS patients with AF was reported (*r* = −0.326, *p* = 0.0147) (92).

##### 3.5.1.2 AIS vs. stroke mimics

Two studies showed higher BNP levels in AIS patients compared to stroke mimics (50, 51). Only one of the studies showed no difference in BNP levels between AIS and stroke mimics (42). BNP was not predictive for AIS (OR: 2.17, IQR: 0.90–5.24, *p* = 0.084) (51).

#### 3.5.2 NT-proBNP

In contrast, three studies measured NT-proBNP and included 1,028 AIS patients and 280 controls (two studies with age- and sex-matched HC, and one study with stroke mimics) (32, 33, 55). Studies were published between 2005 and 2017 and carried out in Greece, Spain, and Turkey. The time of blood sampling was from within <6 h to within <24 h after symptom onset. NT-proBNP concentrations were measured with electrochemiluminescence systems, enzyme immunoassay, and an antibody-based array (Table 6). The risk of bias assessment revealed good quality in one study and moderate quality in two studies.

All studies reported higher NT-proBNP levels in patients with AIS compared to controls. Additionally, AIS patients with an infarct diameter > 3 cm showed elevated NT-proBNP concentrations when compared to controls (logNT-proBNP: 7.96 ng/mL, SD: 1.66 vs. 6.52 ng/mL, SD: 1.6, *p* = 0.002) (33). However, AIS patients with electrocardiogram changes had higher NT-proBNP levels compared to those without changes, especially, patients with impaired left ventricular ejection fraction as well as impaired left ventricular end-diastolic diameter (*p* = 0.019, *p* = 0.011) (33). Interestingly, NT-proBNP levels did not differ whether strokes occurred in the carotid or vertebrobasilar location (32). No correlation between stroke severity or infarct size was observed (32). However, on the day of admission, NT-proBNP concentration between CEI and atherothrombotic-caused stroke differed (166.3 fmol/mL, SEM: 25.3 vs. 108.4 fmol/mL, SEM: 8.3, *p* ≤ 0.05) (32). In a study of 941 AIS patients and 193 stroke mimic patients, a ROC analysis at a cut-off value of 4.685 pg./mL showed a sensitivity of 76.9% and a specificity of 43.5% (AUC = 0.742, 95% CI: 0.686–0.797, *p* < 0.0001) (55).

### 3.6 Biomarker panels

In addition to the examination of individual biomarkers, eight studies created panels to predict AIS. Those studies were published between 2003 and 2014 (Table 7) (34, 42, 46, 50, 53, 78, 90, 91).

**Table 7:** Biomarker panels.

Analysis	Author	Study type	Study year	Study period	Origin of the study	Blood drawing in h	Measurement method	AIS (N)	AIS age median [IQR] mean (SD)	AIS female/male (N)	Definition controls	Control group (N)	Control age median [IQR] Mean (SD)	Control female/male (N)	Outcome
Controls other than stroke mimics															
vWF + MMP-9 + S100B + MCP-1 + BNGF	Reynolds et al.	Prospective case–control	2003	11/1999–02/2003	USA	12	ELISA	54	n.a.	n.a.	HC	214	n.a.	n.a.	*ROC-analysis* (<12 h): AUC: n.a. (sensitivity: 0.907, specificity: 0.967)
MMX = BNP + D-dimer + MMP-9 + S100B	Kim et al.	Prospective case–control	2010	04/2007–08/2007	South Korea	6	Triage^®^ Meter POCT instrument (Biosite, Inc.).	89	66.6 (11.8)	39/50	HC	57	43.8 (12)	32/25	AIS: 4.0 (1.9) vs. HC: 2.1 (1.1), *p* < 0.001
IMA, S100B, NSE	Cakmak et al.	Prospective case–control	2014	n.a.	Turkey	9.2 (9)	ELISA	38	66.1(12.8)	18/20	Control group including TIAs	30	64.6 (12.4)	10/20	*Sensitivity*: IMA + NSE (0.95); IMA + S100B (0.97); NSE + S100B (0.92); IMA + NSE + S100B (0.97) *Specificity:* IMA + NSE (0.3); IMA + S100B (0.37); NSE + S100B (0.47); IMA + NSE + S100b (0.23)
Stroke mimics															
BNP, D-dimer, MMP-9, S100B	Laskowitz et al.	Multicenter prospective cohort study	2009	07/2002–06/2005	USA	9.3 [4.5–18.2]	Point-of-care platform (POCT)	293	n.a.	n.a.	Stroke mimics	361	n.a.	n.a.	Log model for differentiation AIS and stroke mimic (*c* = 0.67), At 0–3 h (*c* = 0.73), At 3–6 h (*c* = 0.61), At 6–12 h (*c* = 0.71), At 12–24 h (*c* = 0.66) *Logistic regression*: Sensitivity: 27–91% (75th and 25th percentile), Specificity between 45 and 89% (25th and 75th percentile). *Predictive probability model*:
															AIS vs. Non-AIS OR = between 1 (1st Quartile ≤ 0.38) and 5.23 (4th Quartile ≥0.62), significant from 3rd Quartile (0.48–0.62) on, *p* < 0.0001
BNP, Chimerin II, D-dimer, MMP-9, sRAGE, Secretagogin	Montaner et al.	Prospective, observational	2010	n.a.	Spain	24	ELISA	915	72.63 (12.46)	443/471	Stroke mimics	90	69.57 (17.13)	34/65	*ROC-analysis:* AUC: 0.768 (95%CI: 0.715–0.820)
MMX = S100B + MMP-9 + BNP + D-dimer	Vanni et al.	Prospective, observational	2011	06/2006–09/2006	Italy	6 (7)	Triage^®^ Meter point-of-care instrument	87	74 (11)	34/53	Stroke mimics	68	69 (16)	31/38	MMX AIS: 5.1 (1.9) vs. MMX Stroke mimics: 3.5 (0.9), *p* < 0.001 *ROC-analysis*: *Biomarker alone*: *at <3 h*: 0.72 (95% CI: 0.56–0.82; *p* < 0.001), *between 3–6 h*: 0.85 (95% CI: 0.67–0.94; *p* < 0.001), *>6 h*: 0.68 (95% CI: 0.52–0.81. *p* = 0.026) *Biomarker + clinical features:* 0.86 (95% CI: 0.79–0.91) - > compared to biomarker alone, *p* < 0.01
S100B, IL-6, MMP-9	An et al.	Prospective, observational	2013	09/2010–10/2010	South Korea	11 [7–16]	ELISA	188	66 (11)	87/101	Stroke mimics	90	61 (9)	53/37	*Logistic regression model including*: age, Afib, FAST, logIl-6, logS100B, and logMMP-9: *ROC-analysis:* 0.865 vs. 0.837, *p* = 0.069
H-FABP + S100B	Park et al.	Prospective, observational	2013	09/2008–12/2010	South Korea	11.4 (9.7)	ELISA	111	n.a.	n.a.	Stroke mimics	127	63 (9)	77/50	*ROC-analysis:* AUC: 0.75 (sensitivity: 0.668, specificity: 0.732)

Afib, Atrial fibrillation; TIA, Transient Ischemic Attack; AIS, Acute Ischemic Stroke; h, hours; N, Number of patients; SD, Standard deviation; SEM, Standard Error of the Mean; IQR, Inter Quartile Range; n.a., not applicable; AUC, Area Under Curve; ROC, Receiver Operating Characteristic; HC, Healthy Controls; ELISA, Enzyme-linked Immunosorbent Assay; FAST, Face-Arm-Speech Test; IMA, Ischemia Modified Albumin; NSE, Neuron Specific Enolase; MMP-9, Matrix metalloproteinase 9; BNP, Brain Natriuretic Peptide; MMX, Multi Marker Index; hFABP, high Fatty Acid Binding Protein; IL-6, Interleukin 6; sRAGE, soluble Receptor for Advanced Glycation End Products; MCP-1, Monocyte Chemotactic Protein-1; BNGF, Beta Nerve Growth Factor; vWF, von Willebrand factor.

One study investigated 293 AIS patients and 361 stroke mimics, some evaluated with brain CT lesions and some without. The median time from ‘last seen normal’ to blood draw was 9.3 h (IQR: 4.5–18.2) (91). A logistic regression model including the predictors BNP, MMP-9, S100B, and D-dimer provided modest discriminative capacity (c-statistics = 0.67). The highest capacity was achieved at 3 h after symptom onset (*c* = 0.73), and still considered modest. OR were reported for the 3rd and 4th quartiles of this panel (Q3: 0.48–0.62; Q4: >0.62, *p* < 0.0001). OR ranged between 3.18 and 5.23 evaluated by probability quartiles. Additionally, the NIHSS correlated positively with logistic regression values (*r* = 0.431, *p* < 0.0001). For the detection of stroke within 3 h, logistic regression analyses showed a sensitivity ranging between 27 and 91% (75th and 25th percentile) and a specificity between 45 and 89% (25th and 75th percentile). BNP concentration contributed the most and, S100B, the least. However, the panel showed a positive correlation with ischemic lesions on CT (*p* ≤ 0.05) (91).

Another study investigated MMP-9, BNP, S100B, and D-dimer. The derived multimarker index level was higher in AIS patients compared to stroke mimics (50). Evaluating single biomarkers, it was shown that D-dimer and BNP differed whereas S100B and MMP-9 showed conflicting results. The best cut-off value for this panel was determined to be an index of 4.5, with a PPV and NPV of 0.76 (95% CI: 0.68–0.83) and 0.61 (95% CI: 0.54–0.67), respectively. A ROC curve was generated which showed an AUC of 0.85 (95% CI: 0.67–0.94) when the blood draw was performed <3 h (*p* < 0.001) after symptom onset. Later measurements were less robust [AUC: 0.68 (95% CI: 0.52–0.81), *p* = 0.026]. The combination of the biomarker panel index and stroke severity measured by the Cincinnati Prehospital Stroke Scale enhanced the ROC analysis results [0.86 (95% CI: 0.79–0.91), *p* < 0.01] (50).

Another biomarker panel included NSE, S100B, and ischemia-modified albumin. Using these combined biomarkers, a sensitivity of 97% was achieved however specificity was found to be 23% with an NNPV of 88% and a PPV of 62% for discrimination of AIS versus stroke mimics. Combining the most promising biomarkers in the panel, i.e., ischemia-modified albumin and S100B gave the best results with a sensitivity of 97%, specificity of 37%, NPV of 66%, and a PPV of 92% (78).

A study of 188 AIS patients and 90 stroke mimics reported on a biomarker panel including IL-6, S100B, and MMP-9 which was incorporated in a clinical data assessment (age, AF, Face-Arm-Speech-Test results). It was shown that ROC analysis did not yield better results if biomarkers were added to the analysis (AUC: 0.865 vs. 0.837 with biomarkers, *p* = 0.069). Interestingly the clinical assessment showed better results compared to the biomarkers alone (AUC: 0.837 vs. 0.749, *p* = 0.017) (53).

Another study investigated 915 AIS patients and 90 stroke mimics and reported that the best sensitivity and specificity calculated by ROC analysis were found for caspase-3, D-dimer, soluble Receptor for Advanced Glycation End Products (sRAGE), Chimerin-II, secretagogin, and MMP-9, which formed the basis of the biomarker panel. Calculated ROC including all six biomarkers revealed that if caspase-3, d-dimer, and sRAGE were high and the other biomarkers were low, a stroke probability of 100% was predicted. However, discrimination of ischemic strokes from stroke mimics with blood sampling <24 h from symptom onset, illustrated that the AUC was moderate [0.759 (95% CI: 0.705–0.813)] (42).

A biomarker panel examined among 89 patients with stroke and 38 HC consisting of BNP, D-dimer, MMP-9, and S100B, and a derived multimarker index, showed higher values in stroke patients compared to HC. An AUC of 0.714 was calculated for the diagnosis of AIS. When applying a cut-off value of 1.3, sensitivity was 91%, and specificity 21.5%. However, when applying the upper quartile cut-off value of 5.9, sensitivity slightly increased whereas specificity sharply decreased (sensitivity: 93.5%, specificity: 5.9%) (34).

A biomarker panel comprised of S100B, and high fatty acid binding protein (H-FABP) was conducted among 111 AIS patients, and 127 stroke mimics. However, individually, each of them showed moderate diagnostic value referring to the ROC analysis (H-FABP: AUC: 0.71, cut-off value: 9.7 ng/mL, sensitivity: 59.5%, specificity: 79.5%; S100B: AUC: 0.70, cut off value 23.5 pg/mL, sensitivity: 54.0%, specificity: 83.5%). The combination of the two biomarkers did not increase the diagnostic value (AUC: 0.75, sensitivity: 66.8%, specificity: 73.2%) (90).

A biomarker panel conducted among 54 AIS patients and 214 stroke mimics that included a combination of von Willebrand factor, MMP-9, S100B, Monocyte chemoattractant protein-1, B-type neurotrophic growth factor yielded a sensitivity of 90.7%, and a specificity of 96.7% for detection of ischemic stroke within 12 h (46).

## 4 Discussion

Stroke remains the second most common cause of death worldwide, with an incidence of approximately 15 million patients experiencing AIS every year. A rising trend from 1990 to 2019 was observed, which may be linked to increasing prosperity, rising life expectancy, improved medical care, and new therapies for once-incurable communicable and non-communicable diseases (95, 96). For example, people with HIV, have a life expectancy similar to those without HIV when receiving the best medical care (97, 98). To adequately meet the challenge of the increasing number of strokes globally and global variation in stroke subtypes, we need tools that enable reliable indication of a stroke.

We conducted a systematic review of 13 blood-based protein biomarkers for the diagnosis of AIS, from 61 studies that collected biomarker data within 24 h of symptom onset. The major strength of this review is that we focused on the predictive value of blood-based protein biomarkers for the diagnosis of AIS across a variety of pathologic mechanisms including neurovascular inflammation (MMP-9, TNF-alpha), endothelial integrity (VCAM-1, ICAM-1), cell migration (E-Selectin, P-Selectin, L-Selectin), and glial and astrocytic neuronal biomarker (NSE, GFAP, S100, S100B), and cardiac dysfunction (BNP, NT-proBNP). S100, P-Selectin, and BNP showed overall positive results in differentiating AIS from matched controls and HC (34, 50, 51, 61, 71, 73, 74, 87, 89, 92, 93).

Interestingly, BNP was the only blood-based protein biomarker that showed good differentiability when comparing AIS to stroke mimics which might be partly explained by a high cardiac burden in AIS (50, 51). However, it should be considered that AIS patients may already have cardiovascular risk factors or have even suffered a cardiovascular event, which may have led to pre-existing higher BNP values. Preexisting AF or hypertension is accompanied by higher BNP levels which is reflected by the results of some of the included studies herein (99–102). This also suggests that stroke mimics better reflect reality, as they show stroke-like symptoms, especially in the acute phase, making it difficult to distinguish them from AIS patients. However, only 26.4% of studies examined stroke mimics as a control group. This represents a major limitation related to generalizability. It is not the healthy patient who is admitted to the hospital, but symptomatic patients in whom a stroke is suspected. It is of utmost importance to include a cohort of stroke mimics, as it is often challenging to differentiate them using acute diagnostics. For example, some studies investigating VCAM-1, ICAM-1, and P-Selectin, only examined healthy or matched controls, and most studies were positively predictive for AIS (60–62, 64–68, 70). All studies on P-Selectin showed significant differences in protein levels between AIS patients and controls (61, 62, 71). However, no stroke mimics were investigated. Unfortunately, this approach has not been pursued for VCAM-1, ICAM-1, and P-Selectin since 2013.

Nevertheless, comparisons of AIS patients with stroke mimics should be treated with caution for several reasons. Firstly, in all studies that had stroke mimics as a control group, blood samples were only taken at one point in time and therefore possible short-term changes in protein levels were not represented. Secondly, unmapped kinetics of blood protein levels could potentially provide additional information and allow differentiation between AIS and stroke mimics. This is of great importance, as patients with AIS can, according to current guidelines, only benefit from systemic lysis therapy within 4.5 h or, under certain circumstances, from endovascular therapy within 24 h (7, 103–105). Thirdly, there are only four published studies that have investigated AIS and stroke mimics in the last 5 years (15, 16, 57, 84).

Further limitations for the generalizability of published studies lie in the high heterogeneity of these studies. First, studies included in this review show a high geographical variability. Of 61 studies, 20 countries were represented, however, there is only one Low and Middle Income Country represented for which blood-based biomarkers are important given the scarcity of brain imaging (106, 107). Therefore, regional variations in race and ethnicity, diagnostic criteria and brain imaging modalities, access to healthcare generally, and access to and degree of technology for biomarker measurements, in addition to other differences are important for the interpretation of these data. This must also be considered for future studies, as possible race/ethnic differences may contribute to different protein kinetics in the acute phase, and thus may have affected the results in the studies we included. Second, all the evaluated blood-based protein biomarkers are not exclusively observed in the condition of stroke. For example, elevated S100B levels are also observed in patients with multiple sclerosis, amyotrophic lateral sclerosis, or cancer, as S100B is found in all neuronal tissue thus leading to a low specificity (108–111). This is also the case with GFAP and NSE and although BNP is a cardiac peptide, it can also be elevated in diseases that cause secondary cardiac stress (e.g., pulmonary hypertension, stroke, renal insufficiency) (112–118).

GFAP is one of the most studied biomarkers in stroke but is also broadly investigated in differentiating AIS from intracerebral bleeding (15, 16, 119). However, few studies dealt with the diagnostic value of GFAP in AIS patients within the first 24 h (15, 120). The majority of studies included in this analysis showed higher GFAP levels in AIS patients compared to HC/matched controls but not to stroke mimics, possibly due to its low specificity. Elevated GFAP concentrations can also be found in multiple sclerosis, brain tumors, encephalitis, amyotrophic lateral sclerosis, and Alzheimer’s Disease which was reflected by an AUC ranging between 0.67 and 0.71 and a maximum specificity of 70% (116–118, 121).

Additionally, we searched included studies on whether developed biomarker panels were eligible for AIS discrimination (Table 7). Investigated panels consisted of different combinations of MMP-9, BNP, D-dimer, NSE, ischemia-modified albumin, S100B, and HFABP. A promising panel included S100B, MMP-9, BNP, and D-dimer, which, in combination with the clinical characteristics of the patients, achieved a high AUC (50). However, the individual biomarkers did not perform well on their own and their specificity was rather low. Nevertheless, this approach may be the most promising compared to other panels studied, which were rather unspecific (42, 90, 91). Other panels, which also examined stroke mimic patients without taking clinical characteristics into account, were found to have low specificity (34, 42, 53, 78, 90, 91).

Overall, blood-based protein biomarkers may be value-added for acute diagnostics in patients with AIS in the future. However, there remains a lack of systematic, large-scale studies investigating the prognostic value of blood-based protein biomarkers. Many issues remain. First, there has been a lack of inclusion of stroke mimics, and blood-based protein biomarker kinetics in 21st century published studies. Second, comprehensive standardized global multicenter studies are needed. Third, a further limitation of the included studies is the lack of ethnic representation. Recent studies have demonstrated an additional benefit of incorporating the variable of race. For instance, the implementation of race-adjusted scales was demonstrated to markedly enhance the predictability of disease classification, occupational eligibility, and disability compensation in patients undergoing spirometric testing (122). A comparable impact may also be observed with regard to the predictive accuracy of individual biomarkers, which should be subjected to further investigation in the future. Only two of the included studies presented data on different entheses, therefore no conclusions could be drawn in this review. Fourth, blood-based biomarkers for central nervous system disorders may be elevated in peripheral nervous system disorders, e.g., peripheral neuropathies, trauma, and infectious diseases such as HIV. Fifth, another limitation of this review is the paucity of data regarding the specificity and sensitivity of the biomarkers included in this study. Consequently, a sufficient comparative presentation was not possible. Sixth, ultrasensitive assays, such as SIMOA are expensive and not universally available, nor are all protein assays available in individual clinic settings. Despite this, technical developments will bring this into the clinic at affordable prices. Finally, the measurement of blood-based protein biomarkers is less expensive than brain imaging techniques such as brain CT or brain MRI.

The present review has limitations. It is possible that studies were overlooked in the literature search. To minimize this, we examined reference lists of the included studies as well as other systematic reviews and meta-analyses. Also, we extracted values for blood-based protein biomarkers that were not shown in the text or diagrams using the WebPlotDigitizer, which may have led to bias (38). Nonetheless, to the authors’ knowledge, this represents the most comprehensive systematic review of blood-based protein biomarkers for stroke: MMP-9, TNF-alpha, VCAM-1, ICAM-1, E-Selectin, P-Selectin, L-Selectin, NSE, GFAP, S100, S100B, BNP, and NT-proBNP.

## 5 Conclusion

In summary, the blood-based protein biomarker studies examined in this review do not provide sufficient evidence to assist in the diagnosis of AIS. This is due to the limitations of the studies themselves, as well as inadequate comparability between studies, the lack of studies with stroke mimics as controls, and available protein kinetic changes in the acute AIS phase. Therefore, not only biomarkers such as BNP, S100B, and P-Selectin (i.e., which have shown promise in this review), but also biomarkers such as MMP-9, TNF-alpha, VCAM-1, ICAM-1, E-Selectin, L-Selectin, NSE, GFAP, S100, and NT-proBNP should be further investigated. Considering repeated measurements to map the protein kinetic changes over time in the acute setting (<24 h), and inclusion of stroke mimics to reflect the real-world scenario would enhance clinical diagnostics.

## Data availability statement

The original contributions presented in the study are included in the article/Supplementary material, further inquiries can be directed to the corresponding author.

## Author contributions

JR: Conceptualization, Data curation, Formal analysis, Investigation, Methodology, Project administration, Software, Validation, Visualization, Writing – original draft, Writing – review & editing. AC: Data curation, Investigation, Validation, Writing – review & editing. AS: Validation, Visualization, Writing – review & editing. FB: Conceptualization, Funding acquisition, Methodology, Supervision, Validation, Writing – review & editing. DG: Conceptualization, Funding acquisition, Supervision, Validation, Writing – review & editing. AB: Conceptualization, Funding acquisition, Supervision, Validation, Writing – review & editing.

## Glossary

AIS: Acute ischemic stroke
AF: Atrial fibrillation
AUC: Area under curve
BNP: Brain natriuretic peptide
CEI: Cardio embolic infarcts/stroke
CT: Computer tomography
ELISA: Enzyme-linked immunosorbent assay
Elecsys: Electrochemiluminescence systems
EVT: Endovascular therapy
GCS: Glasgow Coma Scale
GFAP: Glial fibrillary acid protein
HC: Healthy controls
ICAM-1: Intercellular adhesion molecule-1
IQR: Interquartile range
LAC: Lacunar stroke
LVO: Large vessel occlusions
MCA: Middle cerebral artery
MMP-9: Matrix-metalloproteinase 9
MRI: Magnetic resonance imaging
NIHSS: National Institutes of Health and Stroke Scale
NOS: Newcastle-Ottawa Scale
NPV: Negative predictive value
NSE: Neuron-specific enolase
NT-proBNP: N-terminal pro-brain natriuretic peptide
ODE: Other determined etiology
OR: Odds ratio
PACI: Partial anterior circulation infarction
POCT: Point-of-care test device
PPV: Positive predictive value
PRISMA: Preferred reporting items for systematic reviews and meta-analyses
ROC: Receiver operating characteristic
SD: Standard deviation
SEM: Standard error of the mean
SIMOA: Single molecule array
sRAGE: Soluble receptor for advanced glycation end products
TACI: Total anterior circulation infarction
TNF-alpha: Tumor necrosis factor-alpha
VCAM-1: Vascular cell adhesion molecule-1
