# Supplementary material for: Blood-based protein biomarkers during the acute ischemic stroke treatment window: a systematic review
Source: Front Neurol. 2024 Jul 18;15:1411307. doi: 10.3389/fneur.2024.1411307 (PMC11291248; doi:10.3389/fneur.2024.1411307)
Supplement: Supplementary file 1 [file Table_1.docx]

***sTable 1)*** ***Inflammation biomarkers****, MMP-9 Matrix Metalloproteinase-9, TNF Tumor Necrosis Factor, Study quality: ≥7 stars were considered as “good-quality”, between 2 and 6 stars rated studies were considered as “fair-quality”, and ≤1 point was considered as “poor-quality” (Desyibelew and Dadi, 2019; Fekadu Dadi, Miller and Mwanri, 2020; Mengist et al., 2021).*

| **Author** | **Study Year** | **Selection 1** | **Selection 2** | **Selection 3** | **Selection 4** | **Comparability** | **Exposure Outcome 1** | **Exposure Outcome 2** | **Exposure Outcome 3** | **Total** | **Study quality** |
| --- | --- | --- | --- | --- | --- | --- | --- | --- | --- | --- | --- |
| **MMP-9** |  |  |  |  |  |  |  |  |  |  |  |
| Kim et al. | 2010 | * | * | - | - | - | - | * | - | 3 | 2 |
| Montaner et al. | 2001 | * | * | - | - | ** | - | * | - | 3 | 2 |
| Horstmann et al. | 2003 | * | * | - | * | ** | - | * | * | 7 | 1 |
| Ning et al. | 2006 | * | * | * | * | ** | * | * | - | 8 | 1 |
| Heo et al. | 2003 | * | - | * | * | - | * | * | - | 5 | 2 |
| Montaner et al. | 2003 | * | - | - | - | - | - | * | * | 3 | 2 |
| Vukasovic et al. | 2006 | * | * | - | - | - | - | * | - | 3 | 2 |
| Lehmann et al. | 2015 | * | * | * | * | ** | * | * | * | 9 | 1 |
| Bustamante et al. | 2017 | * | * | * | - | * | * | * | * | 7 | 1 |
| An et al. | 2013 | * | * | - | - | - | - | * | * | 4 | 2 |
| Vanni et al. | 2008 | * | * | * | * | * | * | * | - | 7 | 1 |
| Glickman et al. | 2010 | * | - | - | - | - | - | * | - | 2 | 2 |
| Abdelnaseer et al. | 2017 | * | * | - | - | ** | - | * | - | 5 | 2 |
| Lucivero et al. | 2007 | * | * | - | - | - | - | * | - | 3 | 2 |
| Demir et al | 2012 | * | - | * | - | - | * | * | - | 4 | 2 |
| Li et al. | 2022 | * | * | - | * | - | * | * | * | 6 | 2 |
| Kowalski et al. | 2023 | * | * | - | * | - | - | * | * | 5 | 2 |
| Montaner et al. | 2010 | * | * | * | - | * | * | * | - | 6 | 2 |
| Reynolds et al. | 2003 | * | - | * | - | * | * | * | - | 5 | 2 |
| Castellanos et al. | 2003 | * | * | - | - | - | * | * | - | 4 | 2 |
| Laskowitz et al. | 2009 | * | * | * | * | * | * | * | - | 7 | 1 |
| **TNF** |  |  |  |  |  |  |  |  |  |  |  |
| An et al. | 2013 | * | * | - | - | - | - | * | * | 4 | 2 |
| Licata et al. | 2009 | * | * | * | - | ** | * | * | * | 8 | 1 |
| Intiso et al. | 2004 | * | * | * | * | ** | * | * | * | 8 | 1 |
| Tuttolomondo et al. | 2009 | * | * | * | - | ** | * | * | - | 7 | 1 |
| Lehmann et al. | 2015 | * | * | * | * | ** | * | * | * | 9 | 1 |
| Cevik et al. | 2013 | * | - | - | - | - | - | * | - | 2 | 2 |
| Zaremba et al. | 2001 | * | * | - | - | * | - | * | - | 4 | 2 |
| Sotgiu et al. | 2006 | * | * | * | - | ** | * | * | * | 7 | 1 |
| Musumeci et al. | 2013 | * | * | - | - | - | - | * | - | 3 | 2 |
| Kowalski et al. | 2023 | * | * | - | * | - | - | * | * | 5 | 2 |
