# Supplementary material for: Blood-based protein biomarkers during the acute ischemic stroke treatment window: a systematic review
Source: Front Neurol. 2024 Jul 18;15:1411307. doi: 10.3389/fneur.2024.1411307 (PMC11291248; doi:10.3389/fneur.2024.1411307)
Supplement: Supplementary file 2 [file Table_2.docx]

***sTable 2)*** ***Endothelial integrity biomarkers;*** *VCAM V Vascular Cell Adhesion Molecule 1, ICAM Intercellular Adhesion Molecule 1 Study quality: ≥7 stars were considered as “good-quality”, between 2 and 6 stars rated studies were considered as “fair-quality”, and ≤1 point was considered as “poor-quality” (Desyibelew and Dadi, 2019; Fekadu Dadi, Miller and Mwanri, 2020; Mengist et al., 2021).*

| **Author** | **Study Year** | **Selection 1** | **Selection 2** | **Selection 3** | **Selection 4** | **Comparability** | **Exposure Outcome 1** | **Exposure Outcome 2** | **Exposure Outcome 3** | **Total** | **Study quality** |
| --- | --- | --- | --- | --- | --- | --- | --- | --- | --- | --- | --- |
| **VCAM** |  |  |  |  |  |  |  |  |  |  |  |
| Abdulle et al. | 2010 | * | * | * | - | * | * | * | - | 6 | 2 |
| Fassbender et al. | 1995 | * | - | - | - | - | - | * | - | 2 | 2 |
| Licata et al. | 2009 | * | * | * | - | ** | * | * | * | 8 | 1 |
| Tuttolomondo et al. | 2009 | * | * | * | - | ** | * | * | - | 7 | 1 |
| Supanc et al. | 2010 | * | * | - | * | ** | * | * | - | 7 | 1 |
| Rallidis et al. | 2009 | * | * | - | - | ** | - | * | - | 5 | 2 |
| Musumeci et al. | 2013 | * | * | - | - | - | - | * | - | 3 | 2 |
| Sotgiu et al. | 2006 | * | * | * | - | ** | * | * | - | 7 | 1 |
| Simundic et al. | 2004 | * | - | * | * | - | * | * | - | 6 | 2 |
| **ICAM** |  |  |  |  |  |  |  |  |  |  |  |
| Abdulle et al. | 2010 | * | * | * | - | * | * | * | - | 6 | 2 |
| Fassbender et al. | 1995 | * | - | - | - | - | - | * | - | 2 | 2 |
| Licata et al. | 2009 | * | * | * | - | ** | * | * | * | 8 | 1 |
| Tuttolomondo et al. | 2009 | * | * | * | - | ** | * | * | - | 7 | 1 |
| Supanc et al. | 2010 | * | * | - | * | ** | * | * | - | 7 | 1 |
| Musumeci et al. | 2013 | * | * | - | - | - | - | * | - | 3 | 2 |
| Shyu et al. | 1997 | * | * | - | * | * | * | * | - | 6 | 2 |
| Sotgiu et al. | 2006 | * | * | * | - | ** | * | * | * | 7 | 1 |
| Rallidis et al. | 2009 | * | * | - | - | ** | - | * | - | 5 | 2 |
| Simundic et al. | 2004 | * | - | * | * | - | * | * | - | 6 | 2 |
