# Supplementary material for: Blood-based protein biomarkers during the acute ischemic stroke treatment window: a systematic review
Source: Front Neurol. 2024 Jul 18;15:1411307. doi: 10.3389/fneur.2024.1411307 (PMC11291248; doi:10.3389/fneur.2024.1411307)
Supplement: Supplementary file 4 [file Table_4.docx]

***sTable 4)*** ***Glial and neuronal biomarkers,*** *NSE Neuron Specific Enolase, GFAP Glial Fibrillary Acid Protein, Study quality: ≥7 stars were considered as “good-quality”, between 2 and 6 stars rated studies were considered as “fair-quality”, and ≤1 point was considered as “poor-quality” (Desyibelew and Dadi, 2019; Fekadu Dadi, Miller and Mwanri, 2020; Mengist et al., 2021).*

| **Author** | **Study Year** | **Selection 1** | **Selection 2** | **Selection 3** | **Selection 4** | **Comparability** | **Exposure Outcome 1** | **Exposure Outcome 2** | **Exposure Outcome 3** | **Total** | **Study quality** |
| --- | --- | --- | --- | --- | --- | --- | --- | --- | --- | --- | --- |
| **NSE** |  |  |  |  |  |  |  |  |  |  |  |
| Cakmak et al. | 2014 | * | - | * | * | * | - | * | - | 5 | 2 |
| Missler al. | 1997 | * | - | - | * | - | - | * | - | 3 | 2 |
| Cunningham et al. | 1991 | * | * | - | * | - | - | * | * | 5 | 2 |
| Oh et al. | 2003 | * | - | - | * | ** | - | * | * | 6 | 2 |
| Wu et al. | 2004 | * | * | - | * | - | - | * | - | 4 | 2 |
| Sulter et al. | 1998 | * | - | - | * | - | - | * | * | 4 | 2 |
| Bustamante | 2017 | * | * | * | - | * | * | * | * | 7 | 1 |
| Gojska-Grymajlo et al. | 2018 | * | - | - | * | ** | - | * | - | 5 | 2 |
| An et al. | 2013 | * | * | * | - | - | - | * | * | 5 | 2 |
| Fassbender et al. | 1997 | * | * | * | * | * | * | * | - | 7 | 1 |
| **GFAP** |  |  |  |  |  |  |  |  |  |  |  |
| Ren et al. | 2016 | * | - | - | * | - | - | * | - | 3 | 2 |
| Jaeger et al. | 2023 | * | * | * | - | - | * | * | * | 6 | 2 |
| Katsanos et al. | 2017 | * | * | * | - | * | - | * | - | 5 | 2 |
| Kalra et al. | 2021 | * | * | * | - | * | * | * | * | 7 | 1 |
| Kowalski et al. | 2023 | * | * | - | * | - | - | * | * | 5 | 2 |
| Ferrari et al. | 2023 | * | - | * | * | * | * | * | * | 7 | 1 |
| An et al. | 2013 | * | * | * | - | - | - | * | * | 5 | 2 |
| Luger et al. | 2020 | * | * | * | * | * | * | * | - | 7 | 1 |
| Luger et al. | 2017 | * | * | * | * | * | * | * | - | 7 | 1 |
| Foerch et al. | 2012 | * | - | * | * | * | * | * | - | 6 | 2 |
| Ekingen et al. | 2016 | * | * | - | * | - | * | * | - | 4 | 2 |
| **S100** |  |  |  |  |  |  |  |  |  |  |  |
| Missler et al. | 1997 | * | - | - | * | - | - | * | * | 4 | 2 |
| Üstündağ et al. | 2011 | * | * | * | * | ** | * | * | * | 9 | 1 |
| Fassbender et al. | 1997 | * | * | * | * | * | * | * | - | 7 | 1 |
| Rainer et al. | 2006 | * | * | - | * | - | * | * | - | 5 | 2 |
| Büttner et al. | 1997 | * | * | - | * | - | * | * | - | 5 | 2 |
| **S100B** |  |  |  |  |  |  |  |  |  |  |  |
| Vanni et al. | 2008 | * | * | * | * | * | * | * | - | 7 | 1 |
| Glickman et al. | 2010 | * | - | - | - | - | - | * | - | 2 | 2 |
| Bustamante et al. | 2017 | * | * | * | - | * | * | * | * | 7 | 1 |
| An et al. | 2013 | * | * | - | - | - | - | * | * | 4 | 2 |
| Montaner et al. | 2010 | * | * | * | - | * | * | * | - | 6 | 2 |
| Park et al. | 2012 | * | * | * | - | * | * | * | - | 6 | 2 |
| Cakmak et al. | 2014 | * | - | * | * | * | - | * | * | 6 | 2 |
| Kim et al. | 2010 | * | * | - | - | - | - | * | - | 3 | 2 |
| Reynolds et al. | 2003 | * | - | * | - | * | * | * | - | 5 | 2 |
| Laskowitz et al. | 2009 | * | * | * | * | * | * | * | - | 7 | 1 |
