# Supplementary material for: Blood-based protein biomarkers during the acute ischemic stroke treatment window: a systematic review
Source: Front Neurol. 2024 Jul 18;15:1411307. doi: 10.3389/fneur.2024.1411307 (PMC11291248; doi:10.3389/fneur.2024.1411307)
Supplement: Supplementary file 5 [file Table_5.docx]

***sTable 5)*** ***Biomarkers of cardiac dysfunction;*** *BNP B-Type Natriuretic Peptide, Nt-proBNP N-terminal prohormone of brain natriuretic peptide, Study quality: ≥7 stars were considered as “good-quality”, between 2 and 6 stars rated studies were considered as “fair-quality”, and ≤1 point was considered as “poor-quality” (Desyibelew and Dadi, 2019; Fekadu Dadi, Miller and Mwanri, 2020; Mengist et al., 2021).*

| **Author** | **Study Year** | **Selection 1** | **Selection 2** | **Selection 3** | **Selection 4** | **Comparability** | **Exposure Outcome 1** | **Exposure Outcome 2** | **Exposure Outcome 3** | **Total** | **Study quality** |
| --- | --- | --- | --- | --- | --- | --- | --- | --- | --- | --- | --- |
| **BNP** |  |  |  |  |  |  |  |  |  |  |  |
| Vanni et al. | 2008 | * | * | * | * | * | * | * | - | 7 | 1 |
| Glickman et al. | 2010 | * | - | - | - | - | - | * | - | 2 | 2 |
| Montaner et al. | 2010 | * | * | * | - | * | * | * | - | 6 | 2 |
| Kim et al. | 2010 | * | * | - | - | - | - | * | - | 3 | 2 |
| Saadet Sayan, Dilcan Kotan | 2016 | * | * | - | * | ** | * | * | - | 5 | 2 |
| Nakagawa et al. | 2004 | * | - | - | * | ** | - | * | * | 4 | 2 |
| Cakir et al. | 2010 | * | - | - | - | ** | * | * | - | 5 | 2 |
| Laskowitz et al. | 2009 | * | * | * | * | * | * | * | - | 7 | 1 |
| **NT-pro-BNP** |  |  |  |  |  |  |  |  |  |  |  |
| Giannakoulas et al. | 2005 | * | * | * | * | ** | * | * | - | 8 | 1 |
| Iltumur et al. | 2006 | * | * | - | - | * | * | * | - | 5 | 2 |
| Bustamante et al. | 2017 | * | * | * | - | - | * | * | * | 6 | 2 |
