# Supplementary material for: Blood-based protein biomarkers during the acute ischemic stroke treatment window: a systematic review
Source: Front Neurol. 2024 Jul 18;15:1411307. doi: 10.3389/fneur.2024.1411307 (PMC11291248; doi:10.3389/fneur.2024.1411307)
Supplement: Supplementary file 6 [file Data_Sheet_1.PDF]

Supplementary Material

1. Search Strategy

Medline-Search:

"Stroke"[MeSH Terms] AND ("Proteins"[MeSH Terms] OR “protein”[Title/Abstract] OR “protein-based”[Title/Abstract] OR “protein-based biomarker”[Title/Abstract] OR “protein biomarker”[Title/Abstract] OR “GFAP”[Title/Abstract] OR “glial fibrillary acidic protein”[Title/Abstract] OR “S100”[Title/Abstract] OR “S100B”[Title/Abstract] OR “TNF”[Title/Abstract] OR “tumor necrosis factor”[Title/Abstract] OR “VCAM”[Title/Abstract] OR “Vascular adhesion molecule”[Title/Abstract] OR “ICAM”[Title/Abstract] OR “intercellular adhesion molecule”[Title/Abstract] OR “Selectin”[Title/Abstract] OR “NSE”[Title/Abstract] OR “neuron specific enolase”[Title/Abstract] OR “MMP-9”[Title/Abstract] OR “matrix metalloproteases”[Title/Abstract] OR “BNP”[Title/Abstract] OR “brain natriuretic peptide”[Title/Abstract] OR “proBNP”[Title/Abstract] or “NT-proBNP”[Title/Abstract]) NOT (“MICE”[Title/Abstract] OR “Mouse”[Title/Abstract] OR “rat”[Title/Abstract] OR “rats”[Title/Abstract] OR “animal”[Title/Abstract])

Embase-Search:

Search restricted to the English language, search up to 07th 11/2023, Abstracts are screened, limited to studies involving humans, removing MEDLINE Records

(ischemic stroke or ischaemic stroke) and (protein or protein-based or protein-based biomarker or protein biomarker or GFAP OR glial fibrillary acidic protein OR S100 OR S100B NSE OR neuron specific enolase OR TNF OR tumor necrosis factor OR VCAM OR Vascular adhesion molecule OR ICAM OR intercellular adhesion molecule OR Selectin OR MMP 9 OR matrix metalloproteases OR BNP OR brain natriuretic peptide OR proBNP OR NT-proBNP).ti or (ischemic stroke or ischaemic stroke) and (protein or protein-based or protein based biomarker or protein biomarker or GFAP OR glial fibrillary acidic protein OR S100 OR S100B OR NSE OR neuron specific enolase OR TNF OR tumor necrosis factor OR VCAM OR Vascular adhesion molecule OR ICAM OR intercellular adhesion molecule OR Selectin OR MMP 9 OR matrix metalloproteases OR BNP OR brain natriuretic peptide OR proBNP OR NT-proBNP).ab not (MICE OR Mouse OR rat OR rats OR animal).ti not (MICE OR Mouse OR rat OR rats OR animal).ab

The Web Of Science:

(AB=(("ischemic stroke" or "ischaemic stroke") and (protein or "protein-based" or “protein-based biomarker” or "protein biomarker" or “GFAP” or “glial fibrillary acidic protein” or “S100” or

“S100B” or “NSE” or “neuron specific enolase” or “TNF” or “tumor necrosis factor” or “VCAM” or “Vascular adhesion molecule” or “ICAM” or “intercellular adhesion molecule” or “Selectin” or “MMP-9” or “matrix metalloproteases” or “BNP” or “brain natriuretic peptide” or ”NT-proBNP” or “proBNP”) not (“MICE” or “Mouse” or “rat” or “rats” or “animal”))) or (TI=(("ischemic stroke" or "ischaemic stroke") and (protein or "protein-based" or “protein-based biomarker” or "protein biomarker" or “GFAP” or “glial fibrillary acidic protein” or “S100” or “S100B” or “NSE” or “neuron specific enolase” or “TNF” or “tumor necrosis factor” or “VCAM” or “Vascular adhesion molecule” or “ICAM” or “intercellular adhesion molecule” or “Selectin” or “MMP-9” or “matrix metalloproteases” or “BNP” or “brain natriuretic peptide” or ”NT-proBNP” or “proBNP”) not (“MICE” or “Mouse” or “rat” or “rats” or “animal”)))

Cochrane Library:

Date Run: 07/11/2023 01:27:07
Comment: Human

**ID**	**Search**	**Hits**
#1	MeSH descriptor: [Cerebrovascular Disorders] explode all trees	22975
#2	(protein):ti,ab,kw	98619
#3	(protein based):ti,ab,kw	17481
#4	(protein based biomarker):ti,ab,kw	1286
#5	(protein biomarker):ti,ab,kw	4472
#6	(GFAP):ti,ab,kw	186
#7	("glial fibrillary acid protein"):ti,ab,kw	10
#8	(S100):ti,ab,kw	486
#9	(S100B):ti,ab,kw	382
#10	(NSE):ti,ab,kw	665
#11	(Neuron specific enolase):ti,ab,kw	550
#11	TNF	16984
#12	Tumor necrosis factor	14517
#13	VCAM	1262
#14	Vascular adhesion molecule	1506
#15	ICAM	1740
#16	Intercellular adhesion molecule	1529
#17	Selectin	2235
#18	(MMP-9):ti,ab,kw	1157
#19	(MMP):ti,ab,kw	2324
#20	("matrix metalloproteinase"):ti,ab,kw	1750
#21	("atrionatriuretic"):ti,ab,kw	2
#22	(BNP):ti,ab,kw	6261
#23	("brain natriuretic peptide"):ti,ab,kw	2466
#24	MeSH descriptor: [Mice] explode all trees	2042
#25	(diagnosis):ti,ab,kw	205486
#26	(diagnostic):ti,ab,kw	97997

#27	(acute diagnostic):ti,ab,kw	10091
#28	(diagnosing):ti,ab,kw	2939
#29	#2 OR #3 OR #4 OR #5 OR #6 OR #7 OR #8 OR #9 OR #10 OR #11 OR# 12 OR #13 OR #14 OR #15 OR #16 OR #17 OR #18 OR #19 OR #20 OR #21 OR #22 OR #23	115633
#30	#1 AND #29	1061
#31	#30 NOT #24	1055
#32	#25 OR #26 OR #27 OR #28	270094
#33	#31 AND #32	300
