# Supplementary figures and images for: Blood-based protein biomarkers during the acute ischemic stroke treatment window: a systematic review
Source: Front Neurol. 2024 Jul 18;15:1411307. doi: 10.3389/fneur.2024.1411307 (PMC11291248; doi:10.3389/fneur.2024.1411307)

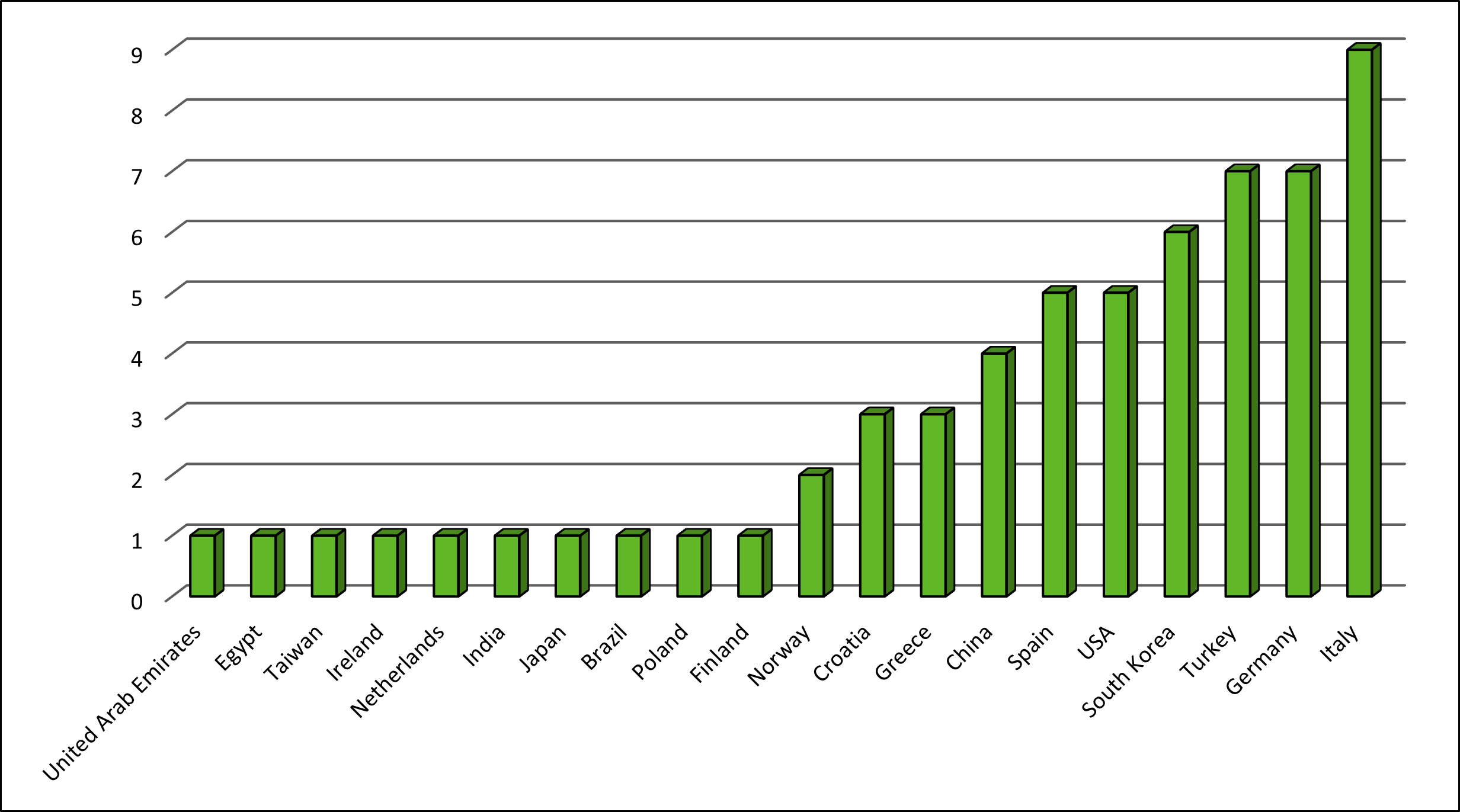

Supplement: Supplementary file 7 [file Image_1.TIF]
